# Homoleptic teflate coordination at platinum(iv): synthesis and characterization of [Pt(OTeF_5_)_6_]^2−^, and covalency trends revealed by the OSLO method

**DOI:** 10.1039/d6sc04436k

**Published:** 2026-08-03

**Authors:** Niklas Limberg, Jacob Mack, Alberto Pérez-Bitrián, André Dallmann, Simon Steinhauer, Sebastian Riedel

**Affiliations:** a Fachbereich Biologie, Chemie, Pharmazie, Institut für Chemie und Biochemie – Anorganische Chemie, Freie Universität Berlin Fabeckstraße 34-36 14195 Berlin Germany s.riedel@fu-berlin.de; b Institut für Chemie, Humboldt-Universität zu Berlin Brook-Taylor-Straße 2 12489 Berlin Germany

## Abstract

The pentafluoroorthotellurate group (teflate, –OTeF_5_) is a unique ligand in transition metal and main group chemistry that combines exceptionally strong electron-withdrawing properties, comparable to those of fluorine, with substantial steric demand. However, its coordination behaviour in transition metal complexes remains poorly understood, in part due to the scarcity of homoleptic transition metal teflate complexes, particularly for late transition metals. By reacting [NEt_4_]_2_[PtCl_6_] and ClOTeF_5_ under solvent-free conditions, we have synthesized the first hexacoordinate homoleptic transition-metal teflate complex, [NEt_4_]_2_[Pt(OTeF_5_)_6_]. This compound exhibits an extraordinary stability: it is bench-stable, resistant toward hydrolysis and alcoholysis, and displays a high thermal decomposition temperature of 200 °C. In the crystallographically determined molecular structure, the O–Te bond lengths, together with the *ν*(O–Te) stretching vibration in the IR spectrum, point toward a pronounced covalent contribution in the Pt–O interaction. To better understand the bonding situation and the electronic stucture of the complex, the system was further analysed using energy decomposition analysis coupled with natural orbitals for chemical valence (EDA-NOCV), quantum theory of atoms in molecules (QTAIM) and the oxidation state localized orbital (OSLO) method. The latter unambiguously assigns an oxidation state of +IV to the platinum centre. A linear correlation between the fragment orbital localization index (FOLI) value of the M–O σ bonding orbital of various homoleptic metal teflate complexes and their experimentally determined mean O–Te bond length was identified. This relationship was subsequently extended to homoleptic main-group teflate compounds, highlighting the general applicability of this approach for assessing the nature of element–teflate bonds.

## Abstract 2

Reflected in the enchanted OSLO mirror, the covalency of homoleptic metal teflate complexes is unveiled: is the newly forged [Pt(OTeF_5_)_6_]^2−^ the fairest? We report its synthesis, structure, multinuclear NMR, and EDA-NOCV, NBO, QTAIM and OSLO analyses.

## Graphical abstract

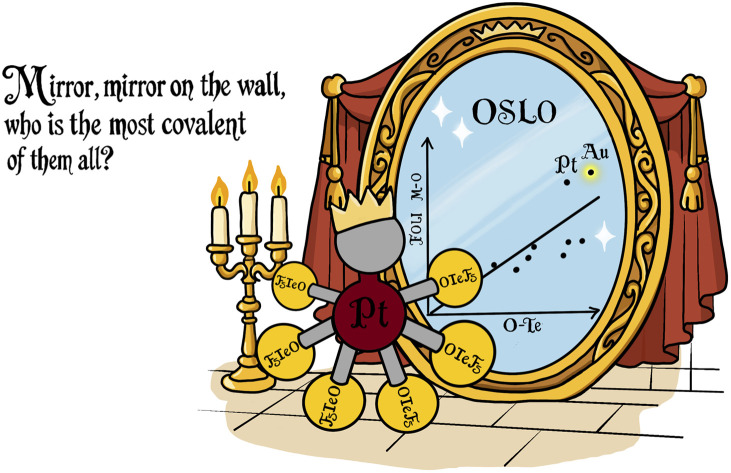

## Introduction

**Scheme 1:**
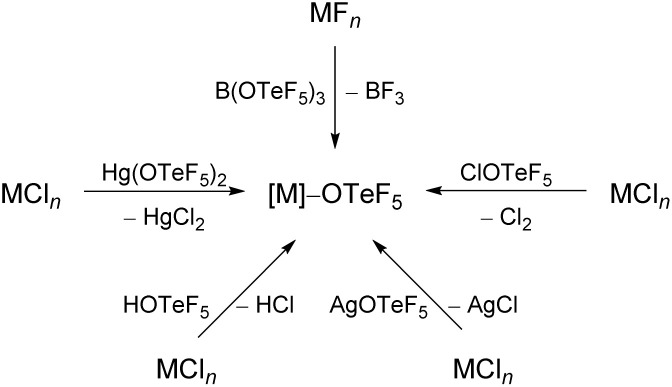
Methods to introduce OTeF_5_ groups into the coordination sphere of transition metals.

Homoleptic transition-metal complexes are fundamental for the development of coordination and organometallic chemistry due to their structural simplicity and their role as well-defined model systems.^1,2^ To elucidate the effect of a mixed coordination sphere around a metal centre, it is essential to first understand simple systems in which all ligands are identical, so that the stereochemistry as well as the chemical and physical properties of a complex are governed solely by the metal–ligand interplay.^2^ However, the synthesis of such complexes often proves challenging, especially for high-valent metal centres coordinated by strongly electron-withdrawing ligands. One ligand system that has received renewed attention is the pentafluoroorthotellurate group (teflate, –OTeF_5_). This unique ligand combines strong electron-withdrawing properties, similar to those of fluorine, with a comparatively pronounced steric demand. Owing to its high degree of charge delocalization and substantial charge-accepting ability, and thus its remarkable robustness towards electrophiles, the teflate ligand enables the formation of weakly coordinating anions and facilitates the stabilization of high oxidation states and highly reactive species.^3,4^ The introduction of the teflate ligand into the coordination sphere of a metal centre is most commonly achieved using metal fluorides or chlorides as precursors in combination with teflate transfer reagents such as AgOTeF_5_, ClOTeF_5_, B(OTeF_5_)_3_, Xe(OTeF_5_)_2_, and HOTeF_5_ (Scheme 1).^3^ Using these approaches, a broad range of homoleptic and heteroleptic transition metal complexes have been prepared. However, to date, only a small number of homoleptic high-valent metal teflate complexes with a coordination number of six are known. These complexes of the general formula [M(OTeF_5_)_6_]^*z*^ (M = Ti, Zr, Hf, Nb, Ta, Mo, W; *z* = 0, −1, −2) are restricted to early- and mid-transition metals. While the complexes of Ti,^5^ Zr,^6^ Hf^6^ and Nb^6–9^ can be prepared as silver salts from the reaction of their metal chloride precursors MCl_4_ (M = Ti, Zr, Hf) and MCl_5_ (M = Nb) with AgOTeF_5_, the corresponding hexacoordinate Ta complex is formed by the reaction of TaCl_5_ with five equivalents of HOTeF_5_ to give the neutral complex, which can be subsequently transformed into its silver salt with AgOTeF_5_.^7,10^ For the homoleptic complexes in the formal oxidation state +VI of W and Mo, their pentachloride precursors, in combination with either Xe(OTeF_5_)_2_ or ClOTeF_5_, yield the desired complexes.^10,11^

**Scheme 2:**
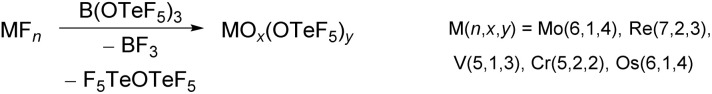
Synthetic route to various oxo containing metal teflates under elimination of F_5_TeOTeF_5_.

For the late transition metals, lower coordination numbers dominate, with four-coordinate being most prevalent, as observed for Co,^12^ Ni,^13^ Pd,^14,15^ Au,^16,17^ Zn,^15^ and Hg.^18^ For Pt, only two teflate-containing complexes have been reported to date, namely Pt(OTeF_5_)_2_(NBD) (NBD = norbornadiene) and Pt(OTeF_5_)_2_(PEt_3_)_2_.^19,20^ One possible explanation for this trend is the propensity of higher-coordinate transition-metal complexes to undergo reductive elimination of F_5_TeOTeF_5_, a process observed for Mo,^10,11^ W,^11^ Re,^10,21^ Os,^10^ Cr,^10^ and V,^10^ which results in the formation of various oxo-containing metal teflate complexes (Scheme 2). This behaviour has hindered a detailed understanding of some fundamental physical and chemical properties associated with the teflate ligand, in particular the nature of metal–teflate bonding in transition metal complexes.

Within this context, we present the synthesis of the first hexa-coordinate late transition-metal complex featuring exclusively M–OTeF_5_ bonds, [NEt_4_]_2_[Pt(OTeF_5_)_6_]. Complementary theoretical analysis using the oxidation state localized orbital (OSLO) method provides insight into the bonding and oxidation state of the platinum centre. With the aim of elucidating metal-teflate interactions and establishing general trends relevant to high-valent transition-metal chemistry, other various homoleptic metal-teflate compounds have been analysed with the OSLO methodology.

## Results and discussion

### Synthesis, characterization and stability of [NEt_4_]_2_[Pt(OTeF_5_)_6_]

**Scheme 3:**
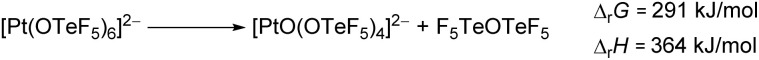
Quantum-chemical investigation of the decomposition channel of [Pt(OTeF_5_)_6_]^2−^ under elimination of F_5_TeOTeF_5_ and formation of the corresponding oxo teflate complex.

Some of the high-valent transition-metal complexes bearing the teflate ligand have been reported to be prone to the elimination of F_5_TeOTeF_5_. However, according to the “oxo wall” concept, such a process leading to the formation of the related oxo complex [PtO(OTeF_5_)_4_]^2−^ would be unexpected due to the anticipated population of metal–oxo π* antibonding orbitals.^22^ To evaluate this possibility, the thermodynamic stability of the complex anion [Pt(OTeF_5_)_6_]^2−^ was assessed by computing the Gibbs free reaction energy (Δ_r_*G*) and the reaction enthalpy (Δ_r_*H*) of the proposed decomposition pathway (Scheme 3). Computational evaluation at the B3LYP/def2-TZVPPD level rendered a Δ_r_*G* of 291 kJ mol^−1^ for this process, indicating a substantial thermodynamic barrier that is expected to ensure stability under ambient conditions.

**Scheme 4:**
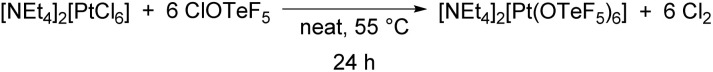
Synthetic route to the homoleptic complex [NEt_4_]_2_[Pt(OTeF_5_)_6_].

To introduce the pentafluoroorthotellurate group to the metal centre, we initially attempted the reaction of the corresponding metal chloride precursor [NEt_4_]_2_[PtCl_6_] with the common teflate transfer reagents AgOTeF_5_ and HOTeF_5_. However, no reaction was observed by ^19^F NMR spectroscopy in any case. Previous studies, including those of our group, have demonstrated the convenience of ClOTeF_5_ as a teflate-transfer reagent for metal centres. In a neat reaction, the teflate group can be transferred under the formation of elemental chlorine due to the partially positively charged chlorine atom in the hypochlorite reagent.^12,13,16,23,24^ Applying this protocol, the reaction of [NEt_4_]_2_[PtCl_6_] and ClOTeF_5_ at 55 °C for 24 hours resulted in the formation of a yellow gas that could be confirmed to be Cl_2_ by UV/Vis spectroscopy (see Fig. S4). Nevertheless, the reaction does not proceed selectively, since a complex mixture was observed by ^19^F NMR spectroscopy. Due to the increasing difficulty of the introduction of the teflate groups in a sequential manner, it is likely that as a byproduct the mixed [PtCl(OTeF_5_)_5_]^2−^ anion is formed. Quantum chemical calculations at B3LYP/def2-TZVPPD (see Table S4) level show the decreasing reaction enthalpy with each additional teflate ligand at the metal centre. While the introduction of the first group has a Gibbs free energy of −135 kJ mol^−1^, it decreases for the last addition to −80 kJ mol^−1^. Despite this trend, the overall Gibbs energy for the introduction of six teflate groups is −612 kJ mol^−1^. The weak-medium field character of the teflate ligand, similar to that of the fluorido,^13^ is underlined by the favoured formation of *cis*-[PtCl_4_(OTeF_5_)_2_] and *fac*-[PtCl_3_(OTeF_5_)_3_] towards the *trans*- and *mer*-complexes due to the higher *trans* effect of the chlorido in comparison to the teflate ligand, as quantum chemical calculations demonstrate (see Table S4). However, washing the crude product with dry EtOH removed the more EtOH-soluble side product, affording the homoleptic complex [NEt_4_]_2_[Pt(OTeF_5_)_6_] (1), which was isolated as a yellow solid (Scheme 4).

**Fig. 1:**
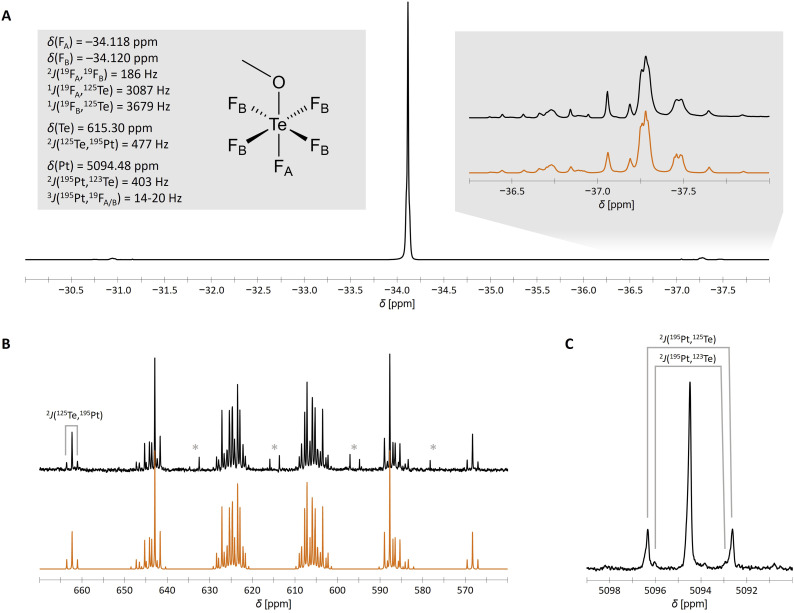
(A) Experimental ^19^F NMR spectrum (564 MHz, *o*DFB, ext. acetone-d_6_, 22 °C) of [NEt_4_]_2_[Pt(OTeF_5_)_6_] with enlarged tellurium satellites (experimental: black; simulation: orange). (B) Experimental (black) and simulated (orange) ^125^Te NMR spectrum (189 MHz, *o*DFB, ext. acetone-d_6_, 22 °C) of [NEt_4_]_2_[Pt(OTeF_5_)_6_], with signals of an unidentified decomposition product marked with asterisk (*). (C) Experimental ^195^Pt{^19^F} NMR spectrum (130 MHz, *o*DFB, ext. acetone-d_6_, 22 °C) of [NEt_4_]_2_[Pt(OTeF_5_)_6_].

Compounds containing the teflate group often give ^19^F NMR spectra of higher order with an AB_4_X or an A_4_BX pattern, where the multiplet patterns can usually be assigned to the chemically and magnetically inequivalent apical (F_A_) or basal (F_B_) fluorine atoms without a more detailed analysis. However, in the case of [Pt(OTeF_5_)_6_]^2−^, the chemical shifts of F_A_ and F_B_ differ only marginally (−34.118 and −34.120 ppm, respectively), and exhibit a typical ^2^*J*(^19^F_A_, ^19^F_B_) coupling constant of 186 Hz, as determined by simulation of the ^19^F NMR spectrum. This very small difference of 0.002 ppm in the chemical shift leads to an overlap of the two signals, resulting in an unusual pattern, in which the expected pseudo doublet and pseudo quintet signals collapse into a pseudo singlet signal (Fig. 1A). A similar effect can be observed, for example, for [Au(OTeF_5_)_4_]^−^ and [Sb(OTeF_5_)_6_]^−^, where *δ*_F_A__ and *δ*_F_B__ are 1.0 ppm and 0.35 ppm apart, respectively.^3,16,25^ The ^125^Te satellites prove particularly useful for the simulation, since their detailed pattern enables a thorough simulation of the coupling constants. Usually, the ^125^Te NMR spectrum of teflate compounds appears as a simple first-order doublet of quintets. When *δ*_F_A__ ≈ *δ*_F_B__, the X part shows severe effects of strong coupling, which is clearly evident in the ^125^Te NMR spectrum (Fig. 1B). An additional ^2^*J*(^125^Te, ^195^Pt) coupling of 477 Hz is observed. The ^195^Pt NMR spectrum shows a singlet with ^125^Te as well as ^123^Te satellites at 5094 ppm (Fig. 1C). This strongly deshielded resonance represents the second most downfield shifted ^195^Pt NMR signal reported to date, surpassed only by [PtF_6_]^2−^ with a resonance of 7330 ppm.^26,27^ This observation can be rationalized within the ligand field framework of Juranić, in which the paramagnetic shielding term scales inversely with the ligand field splitting energy (Δ*E*) and is further amplified by a metal–ligand bond covalency.^26^ The weak-to medium-field character of the teflate ligand, analogous to fluoride,^13^ does not sufficiently increase Δ*E* to effectively reduce the paramagnetic shielding contribution. At the same time, its predominantly σ donating nature leads to comparatively low metal–ligand covalency relative to other ligand systems such as cyanide, chloride, or iodide. The combination of these two effects maximizes the paramagnetic shielding contribution and thereby accounts for the observed extreme downfield shift.

**Fig. 2:**
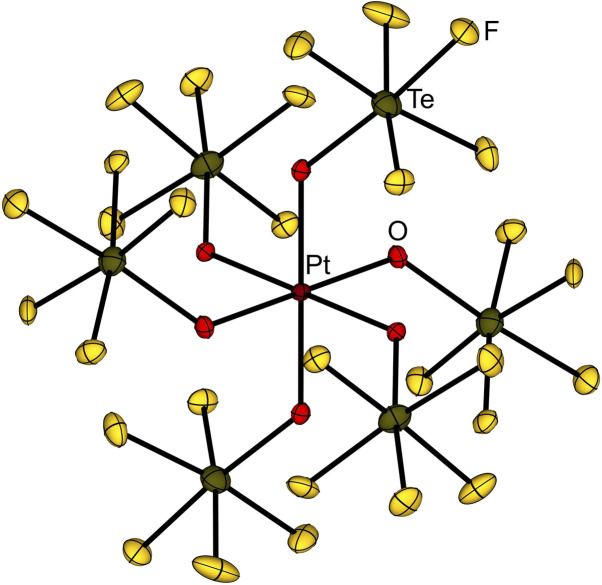
Molecular structure of the [Pt(OTeF_5_)_6_]^2−^ anion in the solid state, as found in crystals of [NEt_4_]_2_[Pt(OTeF_5_)_6_]. One of four crystallographically independent platinum centres coordinated by three pairs of symmetry-related teflate ligands of the asymmetric unit is shown. The [NEt_4_]^+^ cations have been omitted for clarity. Displacement ellipsoids set at 50% probability. Selected mean bond lengths [Å] and mean angles [°]: Pt–O 1.990(10), O–Te 1.829(5), Te–F 1.838(9), Pt–O–Te 131.7(10). For crystallographic details, see SI.

Single crystals of [NEt_4_]_2_[Pt(OTeF_5_)_6_] suitable for X-ray diffraction were grown by careful layering of a concentrated solution of compound 1 in 1,2-difluorobenzene (*o*DFB) with 1,2,3,4-tetrafluorobenzene (4FB) at −20 °C (Fig. 2). Compound 1 crystallizes in the triclinic space group *P*1̄, where the asymmetric unit contains four crystallographically independent platinum centres, each of them coordinated by three pairs of symmetry-related teflate ligands. All four {PtO_6_} cores adopt a nearly ideal octahedral coordination geometry, as reflected by small distortion parameters *Σ* of 4.0–12.8°, where *Σ* is defined as the sum of the absolute deviations of the twelve *cis* O–Pt–O bond angles from the ideal octahedral value of 90° 
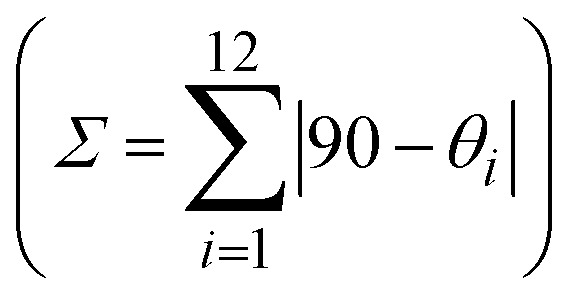
.^28^ The Pt–O bonds range from 1.970(7) to 2.004(7) Å, with a mean Pt–O distance of 1.990(10) Å, being significantly shorter than those observed for the only other known platinum teflate complex Pt^II^(OTeF_5_)_2_(NBD), which exhibits Pt–O bond distances of 2.041(6) and 2.065(5) Å (mean 2.053 Å).^19^ In comparison to other homoleptic Pt(iv) complexes featuring a {PtO_6_} core, the Pt–O bond lengths of 1 are similar to those observed for [Pt(OCOCF_3_)_6_]^2−^ (1.995(4) Å; all Pt–O bonds are symmetry-generated due to rhombohedral space group *R*3̄*c*),^29^ but shorter than those found in [Pt(NO_3_)_6_]^2−^ (2.0065(12)–2.0098(13) Å; mean: 2.0086 Å)^30^ and [Pt(OH)_6_]^2−^ (2.059(2)–2.105(2) Å; mean: 2.079 Å).^31^ The O–Te lengths range between 1.823(9) and 1.838(8) Å (mean: 1.829(5) Å) and are therefore consistent with those reported for other metal teflates such as [Au(OTeF_5_)_4_]^−^ (1.835(4)–1.846(4) Å, mean: 1.840 Å)^16^ or [Ti(OTeF_5_)_6_]^2−^ (1.812(9)–1.822(7) Å, mean: 1.816 Å).^6,8^ However, several other metal teflates, including AgOTeF_5_,^32^ [Co(OTeF_5_)_4_]^2−^,^12^ [Mn(OTeF_5_)_4_]^2−^,^24^ and [Ni(OTeF_5_)_4_]^2−^,^13^ exhibit shorter mean O–Te distances between 1.764–1.790 Å.^12,13,24^

**Fig. 3:**
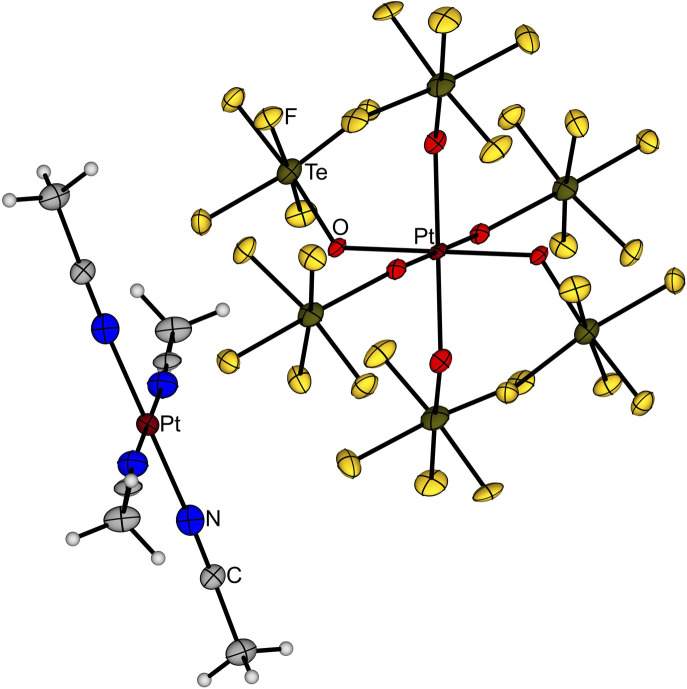
Molecular structure of the [Pt^II^(MeCN)_4_][Pt^IV^(OTeF_5_)_6_] in the solid state. Co-crystallized MeCN has been omitted for clarity. Displacement ellipsoids set at 50% probability. Selected mean bond lengths [Å] and mean angles [°]: Pt–O 1.986(2), O–Te 1.830(6), Te–F 1.835(7), Pt–O–Te 132.3(18). For crystallographic details, see SI.

[NEt_4_]_2_[Pt(OTeF_5_)_6_] exhibits remarkable stability: it is bench-stable and does not decompose upon contact with water or ethanol. This robustness is further underlined by its thermal stability up to 200 °C, as determined by TGA (Fig. S5). However, dissolving the compound in the coordinating solvent acetonitrile leads to partial decomposition, forming the mixed-valence salt [Pt^II^(MeCN)_4_][Pt^IV^(OTeF_5_)_6_] (2) and [NEt_4_][OTeF_5_]. The [Pt(OTeF_5_)_6_]^2−^ anion shows a similar ^19^F NMR signature in MeCN-d_3_ as described before, while [NEt_4_][OTeF_5_] shows a broad doublet at −43.27 ppm and a broad quintet signal at −28.71 ppm, in agreement with literature-reported values.^33^ The formation of 2 was additionally confirmed by single-crystal X-ray diffraction, which reveals distinct platinum cation and anion moieties (Fig. 3). The structure thus features mixed oxidation states at platinum and different coordination environments around the platinum centres. Unfortunately, the mixed-valence product could not be isolated in bulk. Both the cation and the anion are consistent with literature precedents and with the structural metrics discussed above for the [Pt(OTeF_5_)_6_]^2−^ anion.

### Oxidation state localized orbital (OSLO) analysis of homoleptic teflate compounds

The differences in the crystallographically determined O–Te bond lengths observed among several homoleptic metal teflate complexes prompted us to further investigate the origin of these variations. Already in 1985, Strauss and co-workers identified a correlation between the ionicity of the E–OTeF_5_ (E = element) bond and the tellurium–oxygen stretching frequency *ν*(O–Te), the ^19^F NMR chemical shift of the fluorine atom *trans* to oxygen, and the O–Te bond distance. They attributed this effect to a decreasing O-to-Te p–d π bonding contribution when the O-to-E π bonding is increased.^34^ However, this phenomenon can be more appropriately rationalized in terms of negative hyperconjugation, involving donation of the p-type lone pair at the oxygen atom into the σ*(Te–F) orbital, thereby influencing the Te–O bond. This interpretation is consistent with a recent quantum-chemical topology study on XOTeF_5_ systems (X = F, Cl, Br, I), which demonstrates that the internal bonding of the teflate group is primarily ionic in nature and that its electron-withdrawing properties are comparable, albeit slightly lower than, those of fluorine.^35^ In the case of the [Pt(OTeF_5_)_6_]^2−^ system, this donation of the oxygen p-type lone pair, for instance from O5, into the σ*(Te11–F30) orbital results in a stabilisation by 37.9 kJ mol^−1^, as revealed by natural bond analysis (NBO) (Fig. S6). Within the study of Strauss and co-workers, qualitative criteria were proposed, namely a *ν*(O–Te) greater than 820 cm^−1^ and/or a O–Te distance shorter than 1.80 Å, as indicators for a pronounced ionic character of the E–OTeF_5_ bond.^34^ Applying these criteria to compound 1 (*ν*(O–Te) = 796 cm^−1^, O–Te 1.829 Å) already suggests a substantial covalent contribution to the Pt–O bond.

Since the degree of ionicity *versus* covalency of a metal–ligand bond is closely related to the assignment of the oxidation state, a method capable of analysing both simultaneously is particularly valuable. In this regard, the OSLO method offers a unique approach for analysing oxidation states. Compound 1, along with several other homoleptic metal teflate complexes, was therefore analysed using the OSLO method (Table 1).^36^ In addition to the oxidation state, the fragment orbital localization index (FOLI) and the corresponding ΔFOLI values provide key insights. While a FOLI value close to 1 indicates strong localization of an orbital on a defined fragment, a FOLI value approaching 2 reflects significant orbital sharing between two fragments and thus implies increased covalent character. Accordingly, a FOLI value close to 1 is expected for an ionic metal-teflate interaction, while increasing values suggest enhanced covalent M–O bonding. Furthermore, a large ΔFOLI value (the difference between the smallest and second smallest FOLI values of one OSLO orbital) of the last assigned, and therefore least localized orbital, is indicative of an ionic-like orbital assignment, whereas a small ΔFOLI value points toward pronounced covalent sharing.^36^

**Table 1:** Summary of the OSLO analysis together with corresponding experimental relevant mean bond lengths and angles for each compound, as determined by X-ray diffraction studies. The molecular structures were optimized at the B3LYP/def2-TZVPPD level, and the OSLO results were obtained using topological fuzzy Voronoi cell (TFVC) population analysis. DCE = 1,2-dichloroethane

Compound	CCDC	M–O mean [Å]	O–Te mean [Å]	Te–F mean [Å]	M–O–Te mean [°]	FOLI of last OSLO	ΔFOLI of last OSLO	FOLI of σ bond M–O
[Mn(OTeF_5_)_4_]^2−^	2287496	2.020	1.764	1.838	134.0	1.103	3.177	1.083
Ag_2_(OTeF_5_)_2_·2DCE	1181486	2.337	1.781	1.845	129.3	1.092	4.883	1.092
[Co(OTeF_5_)_4_]^2−^	2251373	1.948	1.788	1.848	133.6	1.118	6.909	1.083
[Ni(OTeF_5_)_4_]^2−^	2171818	1.946	1.796	1.842	130.0	1.118	3.040	1.118
[Hg(OTeF_5_)_4_]^2−^	1055055	2.217	1.799	1.844	121.4	1.136	2.809	1.136
[Ti(OTeF_5_)_6_]^2−^	1202467	1.936	1.816	1.840	143.4	1.149	4.912	1.149
[Nb(OTeF_5_)_6_]^−^	1308724	1.930	1.820	1.810	154.7	1.182	3.108	1.182
[Pd(OTeF_5_)_4_]^2−^	1194845	2.002	1.826	1.838	124.3	1.183	3.982	1.183
[Pt(OTeF_5_)_6_]^2−^	2547134	1.990	1.829	1.838	131.7	1.348	1.623	1.348
[Au(OTeF_5_)_4_]^2−^	2156160	1.973	1.840	1.829	120.9	1.358	2.106	1.358

**Fig. 4:**
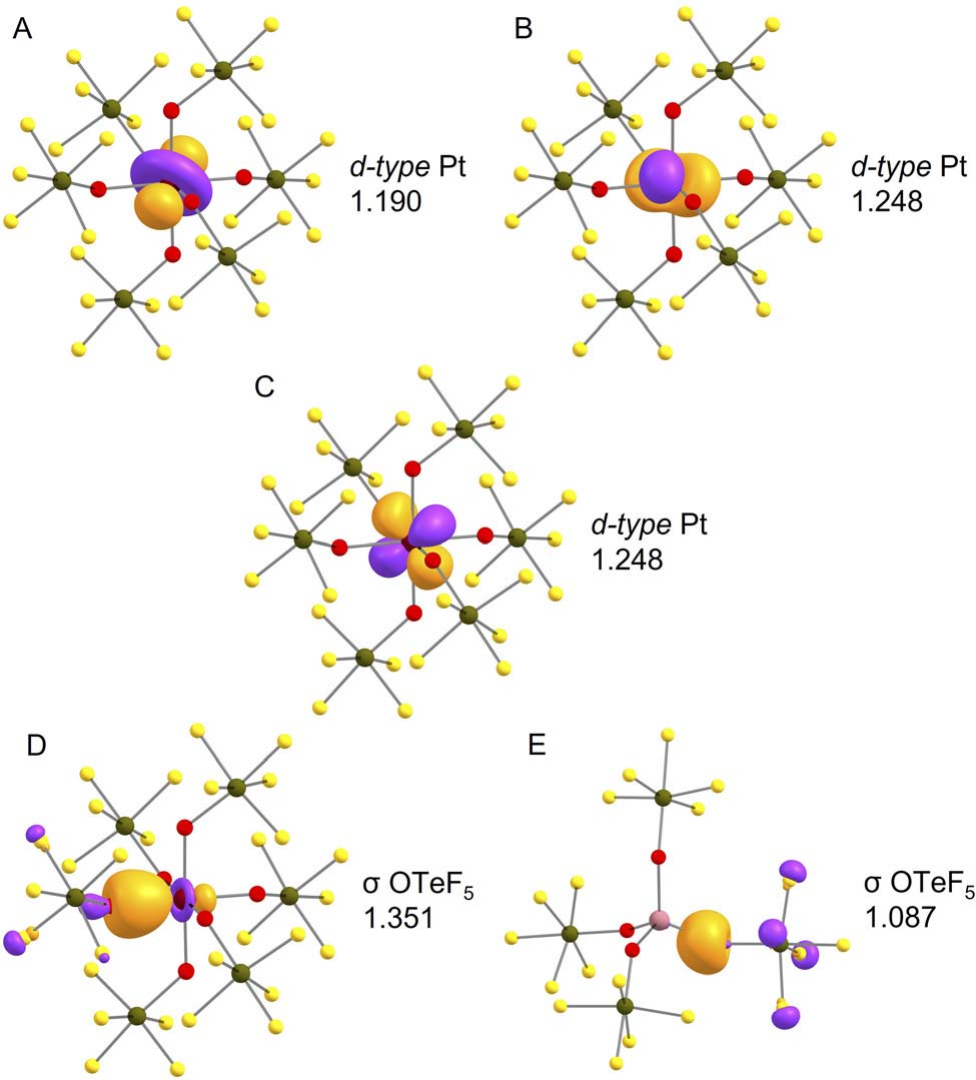
Selected OSLOs of [Pt(OTeF_5_)_6_]^2−^ with TFVC FOLI values for three d-type orbitals at Pt (A–C) and the OTeF_5_ ligand (D). The relatively nonpolar character of the σ Pt–OTeF_5_ interaction is clearly evident. However, the FOLI value (∼1.35) as well as visual inspection indicate that this orbital has greater OTeF_5_ character than Pt (5d) character. OSLO of the OTeF_5_ ligand of complex anion [Co(OTeF_5_)_4_]^2−^ with the corresponding TFVC FOLI value (E). The polar character of the σ Co–OTeF_5_ interaction is clearly evident. The FOLI values, as well as the visual inspection, led to a clear assignment of the respective orbital to either the Co centre or the ligand. The isosurface value for the plots is 0.075 au.

In our study we first started by investigating nine metal teflate complexes, namely [M(OTeF_5_)_4_]^2−^ (M = Mn, Co, Ni, Hg, Pd, Au), [M(OTeF_5_)_6_]^2−^ (M = Ti, Pt), [Nb(OTeF_5_)_6_]^−^ and the dimeric Ag_2_(OTeF_5_)_2_·2DCE (DCE = 1,2-dichloroethane) (Table 1). Indeed, in most investigated cases, the most delocalized orbitals correspond to oxygen-centred p-type orbitals oriented towards the metal centre. Only in three systems, namely AgOTeF_5_, [Co(OTeF_5_)_4_]^2−^, and [Mn(OTeF_5_)_4_]^2−^, metal-centred d-type orbitals constitute the most delocalized orbitals, closely followed by the corresponding M–O bonding orbitals. Comparison of the FOLI values associated with the M–O bonding orbital reveals a range from 1.083 to 1.358, where higher FOLI values indicate increased orbital mixing between the metal and the teflate ligand fragment, and thus a marked variation in bond ionicity. This variation is consistent with the orbital representations shown in Fig. 4, which depicts the M–O σ bonding orbitals of [Pt(OTeF_5_)_6_]^2−^ and [Co(OTeF_5_)_4_]^2−^. Whereas the M–O interaction in [Co(OTeF_5_)_4_]^2−^ (Fig. S8) is predominantly ionic, consistent with previous Interacting Quantum Atoms (IQA) investigations,^12^ the corresponding bond in [Pt(OTeF_5_)_6_]^2−^ exhibits a significant covalent contribution. This is reflected both in the higher FOLI value and in the increased electron density localized at the platinum centre in the corresponding σ bonding orbital. To relate this OSLO based description to established bonding descriptors, NPA charges and QTAIM parameters were additionally evaluated for [M(OTeF_5_)_6_]^2−^ (M = Pt, Ti). Both NPA and QTAIM indicate a polarized bonding situation, with a positively charged Pt centre (NPA: +1.79; QTAIM: +1.81), negatively charged oxygen atoms close to −1, and negatively charged −OTeF_5_ units of approximately −0.63. The QTAIM descriptors nevertheless support a larger shared-interaction component for Pt–O compared with Ti–O, as reflected by the higher electron density at the bond critical point (*ρ*_Pt–O_(*r*) = 0.1230 *vs. ρ*_Ti–O_(*r*) = 0.0974), the more negative total energy density (*H*_Pt–O_(*r*) = −0.0421 *vs. H*_Ti–O_(*r*) = −0.0132), and the larger |*V*|/*G* ratio (Pt–O: 1.2761 *vs.* Ti–O: 1.1040). At the same time, the positive Laplacians for both M–O contacts indicate substantial charge depletion in the nuclear region and thus a pronounced polar contribution. The small M–O ellipticities are consistent with a predominantly σ type interaction.

**Fig. 5:**
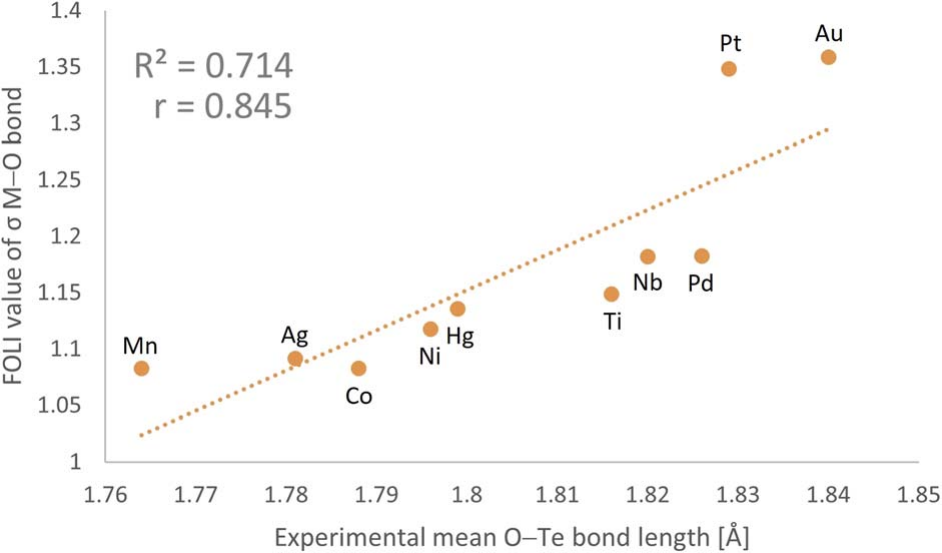
Correlation of the experimental mean bond lengths of the O–Te bond with the FOLI value of the σ M–O bond.

Correlating the FOLI values associated with the σ E–O interaction in various main-group and transition-metal teflate complexes with their respective mean O–Te bond lengths reveals a moderate linear relationship, as indicated by the coefficient of determination (*R*^2^ = 0.682) and the Pearson correlation coefficient (*r* = 0.826) (Fig. S9). However, the correlation seems to be more accurate for small FOLI and bond lengths and deviates for higher FOLI values and bond lengths, which prompted us to analyse main group teflates separately from metal teflates. Indeed, this leads to higher *R*^2^ and *r* values for both correlations indicated by both coefficients (transition metals: Fig. 5, *R*^2^ = 0.714, *r* = 0.845; main group elements: Fig. S10, *R*^2^ = 0.752, *r* = 0.867). In this regard, [Pt(OTeF_5_)_6_]^2−^ fits into this trend and exhibits the highest degree of covalency after [Au(OTeF_5_)_4_]^−^. However, a ΔFOLI value of 1.623 suggests a clear localization at the ligand fragments and thus a clear assignment of the bonding orbitals to the oxygen atom bound to the teflate. Therefore, following the IUPAC^37^ definition for oxidation states, an OS of +IV can be assigned to the platinum centre (Fig. 4). This trend enables a straightforward assessment of bond covalency in metal teflate complexes and main group teflates either *via* experimental bond lengths or through computation of the corresponding FOLI values. This correlation of the M–O FOLI values with the experimental O–Te bond lengths can be rationalised by EDA-NOCV^38^ analyses performed for [Ti(OTeF_5_)_6_]^2−^ and [Pt(OTeF_5_)_6_]^2−^. In both complexes, the orbital interaction energy (*E*_orb_) constitutes the dominant stabilising contribution relative to the electrostatic term (*E*_elstat_) (57% for Ti and 64% for Pt). In each case, *E*_orb_ is clearly dominated by the first NOCV channel, corresponding to σ donation from the ligand to the metal (*E*_orb(1)_ = −32 kcal mol^−1^, |*v*_1_| = 0.51 for Ti; *E*_orb(1)_ = −79 kcal mol^−1^, |*v*_1_| = 0.79 for Pt). The significantly larger eigenvalue and stabilisation energy of this σ channel in [Pt(OTeF_5_)_6_]^2−^ reflects the stronger covalent character of the Pt–O bond, in agreement with its higher FOLI value. Additional π donor contributions (Δ*ρ*_2_ and Δ*ρ*_3_) are present in both complexes but are considerably smaller, with |*v*| values ranging from 0.15 to 0.37, indicating that they play only a secondary role in defining the bonding situation. Importantly, despite their different electronic configurations (d^0^*vs.* d^6^), both systems exhibit the same qualitative bonding pattern, with σ donation as the dominant interaction channel. The differences between the complexes therefore arise primarily from the magnitude, rather than the nature, of the orbital interaction, with the Pt complex showing a stronger overall *E*_orb_ contribution, consistent with its enhanced covalent character. This demonstrates that the d-electron configuration has only limited influence on the bonding mechanism and instead mainly modulates its strength.

Consequently, σ donation emerges as the key factor governing the ionic-to-covalent character of the M–O bond, explaining why d^0^ and d^6^ systems alike follow the same correlation. The FOLI values derived from the OSLO analysis thus provide a reliable and direct measure of bond ionicity across these systems. More broadly, this approach, together with the agreement between OSLO and EDA-NOCV, highlights the robustness of the OSLO method and underscores its potential as a powerful and versatile tool for evaluating bonding situations, which may be extended to other ligand systems.

## Conclusions

In this work, we report the synthesis and characterization of the first homoleptic hexacoordinate metal teflate complex of the late transition metals, [NEt_4_]_2_[Pt(OTeF_5_)_6_]. [NEt_4_]_2_[Pt(OTeF_5_)_6_] could be prepared *via* a neat reaction of [NEt_4_]_2_[PtCl_6_] with ClOTeF_5_ and exhibits a remarkable stability: it is bench stable, resistant toward water and ethanol, and decomposes thermally at temperatures above approximately 200 °C. Upon dissolving in MeCN, partial decomposition is observed, leading to the formation of [Pt(MeCN)_4_][Pt(OTeF_5_)_6_] and [NEt_4_][OTeF_5_]. Single-crystal X-ray diffraction reveals that the [Pt(OTeF_5_)_6_]^2−^ anion adopts a nearly ideal octahedral coordination geometry in the solid state. The observed O–Te bond length, together with the *ν*(O–Te) stretching vibration in the IR spectrum, indicate a pronounced covalent contribution to the Pt–O interaction. This bonding situation was further analysed using energy decomposition analysis coupled with natural orbitals for chemical valence (EDA-NOCV), quantum theory of atoms in molecules (QTAIM) and the oxidation state localized orbital (OSLO) method. EDA-NOCV analysis reveals that the bonding is dominated by ligand-to-metal σ donation, with only minor π contributions, and that both d^0^ and d^6^ systems exhibit the same qualitative interaction pattern, differing primarily in magnitude. A linear correlation between the fragment orbital localization index (FOLI) value of the Pt–O σ bonding orbital and the experimentally determined mean O–Te bond length was identified. This relationship was subsequently extended to main-group teflate complexes, highlighting the OSLO method as a powerful and versatile tool for evaluating bonding situations and may be extended to other ligand systems.

## Author contributions

N. L. designed and performed the experiments, analysed the data, measured and refined the crystal structures, performed quantum-chemical calculations, performed the OSLO analysis, and wrote the manuscript. J. M. performed experiments and quantum-chemical calculations and revised the manuscript. A. D. performed NMR measurements and revised the manuscript. A. P.-B., S. S. and S. R. supervised the project and revised the manuscript.

## Conflicts of interest

There are no conflicts to declare.

## Supplementary Material

SC-OLF-D6SC04436K-s001

SC-OLF-D6SC04436K-s002

## Data Availability

CCDC 2547134 and 2547135 contain the supplementary crystallographic data for this paper.^39*a*,*b*^ Additional details regarding experimental methods and experimental data are given in the supplementary information (SI) and also upon request. Supplementary information is available. See DOI: https://doi.org/10.1039/d6sc04436k.
