# Supplementary material for: Homoleptic teflate coordination at platinum(iv): synthesis and characterization of [Pt(OTeF5)6]2−, and covalency trends revealed by the OSLO method
Source: Chem Sci. 2026 Aug 3;17(39):18635–42. doi: 10.1039/d6sc04436k (PMC13482963; doi:10.1039/d6sc04436k)
Supplement: Supplementary file 1 [file SC-017-D6SC04436K-s001.pdf]

Supplementary Information (SI) for Chemical Science.
This journal is © The Royal Society of Chemistry 2026

Supporting Information

for

Homoleptic Teflate Coordination at Platinum(IV): Synthesis and Characterization of $[Pt(OTeF_5)_6]^{2-}$, and Covalency Trends Revealed by the OSLO Method

Niklas Limberg, Jacob Mack, Alberto Pérez-Bitrián, André Dallmann, Simon Steinhauer and Sebastian Riedel*

Table of Contents

1 Experimental section 3
1.1 General procedures and materials 3
1.2 Synthesis of $[NEt_4]_2[Pt(OTeF_5)_6]$ (**1**) 4
1.3 Synthesis of $[Pt(MeCN)_4][Pt(OTeF_5)_6]$ (**2**) 5
2 NMR spectra 6
3 IR spectra 7
4 UV-VIS spectra 8
5 Thermogravimetric analysis (TGA) 8
6 Crystal data 9
7 Quantum-chemical Analyses 11
8 EDA-NOCV Analysis 12
.... 13
9 OSLO Analysis 14
10 NPA Charges and QTAIM 16
11 References 17

1 Experimental section

1.1 General procedures and materials

All preparative work was carried out using standard Schlenk techniques. Glassware was greased with Triboflon III. $ClOTeF_5$ was prepared from $B(OTeF_5)_3$ and ClF as described elsewhere.[1] $[NEt_4]_2[PtCl_6]$ was prepared from $[NEt_4]Cl$ and $K_2[PtCl_6]$ following a similar method to that described elsewhere.[2] Acetonitrile (MeCN) and ethanol (EtOH) were obtained from an *MBraun* solvent purification system and stored over 3 Å molecular sieves. *ortho*-Difluorobenzene (*o*DFB) and 1,2,3,4-tetrafluorobenzene (4FB) were dried and stored over 3 Å molecular sieves. The moisture content was analysed by Karl Fischer titration with a *Mettler Toledo C10S Coulometric KF Titrator* (<10 ppm). ^{1}H and ^{19}F NMR spectra were recorded on a JEOL 400 MHz ECS, ECZR, ECZLS or on a Bruker Avance NEO 600 MHz spectrometer. ^{125}Te and ^{195}Pt NMR spectra were recorded on a Bruker Avance NEO 600 MHz spectrometer equipped with an ^{1}H-^{19}F-BB triple resonance probe head. All reported chemical shifts were referenced to the Ξ values given in IUPAC recommendations of 2008 using the ^{2}H signal of the deuterated solvent as internal reference.[3] Chemical shifts and coupling constants for strongly coupled spin systems are given as simulated by *gNMR*.[4] IR spectra were measured on a Bruker ALPHA FTIR spectrometer equipped with a diamond ATR attachment in a glove box filled with argon (resolution 4 cm^{-1}). Crystal data were collected with Mo*K*α radiation on a Bruker D8 Venture diffractometer with a CMOS area detector. Single crystals were picked at −40°C under nitrogen atmosphere and mounted on a 0.15 mm Micromount using perfluoroether oil. The structure was solved with the *ShelXT*[5] structure solution program using intrinsic phasing and refined with the *ShelXL*[6] refinement package using least squares on weighted F2 values for all reflections using OLEX2.[7] CCDC 2547134 and CCDC

2547135 contain the supplementary crystallographic data for this paper. These data are provided free of charge by the joint Cambridge Crystallographic Data Centre and Fachinformationszentrum Karlsruhe Access Structures service. The ORCA[8] program was used to perform calculations at the unrestricted Kohn-Sham DFT level, using the B3LYP[9] hybrid functional (with RI) in conjunction with the valence triple-ζ basis set with two sets of polarization functions (def2-TZVPPD).[10] Minima on potential energy surfaces were characterized by normal mode analysis. Thermochemical data is provided without counterpoise correction but including zero-point energy correction as obtained from harmonic vibrational frequencies. The OSLO[11] analysis was performed using the APOST3D[12] package using the topological fuzzy Voronoi cells (TFVC) real-space atomic definition.[13] Therefore, the central atom and each of the corresponding ligands were defined as fragment for the analysis. QTAIM analyses were performed using Multiwfn (version 2026.3.27).[14]

1.2 Synthesis of $[NEt_4]_2[Pt(OTeF_5)_6]$ (**1**)

$ClOTeF_5$ (164 mg, 0.598 mmol, 8 equiv) was condensed at –196 °C onto $[NEt_4]_2[PtCl_6]$ (50 mg, 0.075 mmol, 1 equiv) in a Schlenk tube equipped with a high-vacuum PTFE valve. The reddish reaction mixture was stirred at 50 °C for 24 h. Subsequent removal of all volatiles under reduced pressure at 50 °C for 12 h afforded an orange slurry. The residue was washed with 2 x 0.2 mL of dry EtOH and dried under vacuum to yield the product as a yellow solid (91 mg, 64.5%).

^{19}F NMR (564 MHz, *o*DFB, ext. acetone-d$_6$, 22 °C): δ = –33.12 (F_A, $^2J(^{19}F_A,^{19}F_B)$=186 Hz, $^1J(^{19}F_A,^{125}Te)$=3087 Hz), –33.12 (F_B, $^2J(^{19}F_A,^{19}F_B)$=186 Hz, $^1J(^{19}F_B,^{125}Te)$=3679 Hz) ppm.

^{1}H NMR (400 MHz, *o*DFB, ext. acetone-d$_6$, 22 °C): δ = 1.12 (tt, CH_3, $^3J(^1H,^1H)$=7.3 Hz, $^3J(^1H,^{14}N)$=1.9 Hz), 3.00 (q, CH_2, $^3J(^1H,^1H)$=7.3 Hz) ppm.

^{125}Te NMR (189 MHz, *o*DFB, ext. acetone-d$_6$, 22 °C): δ = 615.3 ($^1J(^{125}Te,^{19}F_A)$=3087 Hz, $^1J(^{125}Te,^{19}F_B)$=3679 Hz, $^2J(^{125}Te,^{195}Pt)$=477 Hz) ppm.

^{195}Pt{^{19}F} NMR (130 MHz, *o*DFB, ext. acetone-d$_6$, 22 °C): δ = 5094.5 ($^2J(^{195}Pt,^{123}Te)$=403 Hz, $^2J(^{195}Pt,^{125}Te)$=477 Hz) ppm.

IR (ATR, 20 °C): $\tilde{\nu}$ = 3002 (w, C–H ν), 1466 (m, C–H δ), 1401 (m, C–H δ), 1186 (m, C–H δ), 1041 (m, C–N ν), 1002 (m, C–C ν), 796 (s, Te–O ν), 697 (s, Te–F ν), 669 (s, Te–F), 624 (m, Te–F), 504 (w, Pt–O) cm^{-1}.

1.3 Synthesis of $[Pt(MeCN)_4][Pt(OTeF_5)_6]$ (**2**)

$ClOTeF_5$ (66 mg, 0.239 mmol, 8 equiv) was condensed at –196 °C onto $[NEt_4]_2[PtCl_6]$ (20 mg, 0.029 mmol, 1 equiv) in a Schlenk tube equipped with a high-vacuum PTFE valve. The reddish reaction mixture was stirred at 50 °C for 24 h. Subsequent removal of all volatiles under reduced pressure at 50 °C for 12 h afforded a yellowish slurry. The residue was dissolved in MeCN and dried under vacuum to yield the product as a yellow solid in a mixture with $[NEt_4][OTeF_5]$.

^{19}F NMR (377 MHz, MeCN-d$_3$, 22 °C): δ = –28.71 (F_A, $^2J(^{19}F_A,^{19}F_B)$=174 Hz, $^1J(^{19}F_A,^{125}Te)$=2914 Hz), –34.52 (F_A, $^2J(^{19}F_A,^{19}F_B)$=174 Hz, $^1J(^{19}F_A,^{125}Te)$=3087 Hz), –34.66 (F_B, $^2J(^{19}F_A,^{19}F_B)$=186 Hz, $^1J(^{19}F_B,^{125}Te)$=3679 Hz), –43.27 (F_B, $^2J(^{19}F_A,^{19}F_B)$=174 Hz, $^1J(^{19}F_a,^{125}Te)$=3586 Hz) ppm.

^{1}H NMR (400 MHz, MeCN-d$_3$, 22 °C): δ = 1.20 (tt, CH_3, $^3J(^1H,^1H)$=7.3 Hz, $^3J(^1H,^{14}N)$=1.9 Hz), 3.15 (q, CH_2, $^3J(^1H,^1H)$=7.3 Hz) ppm.

2 NMR spectra

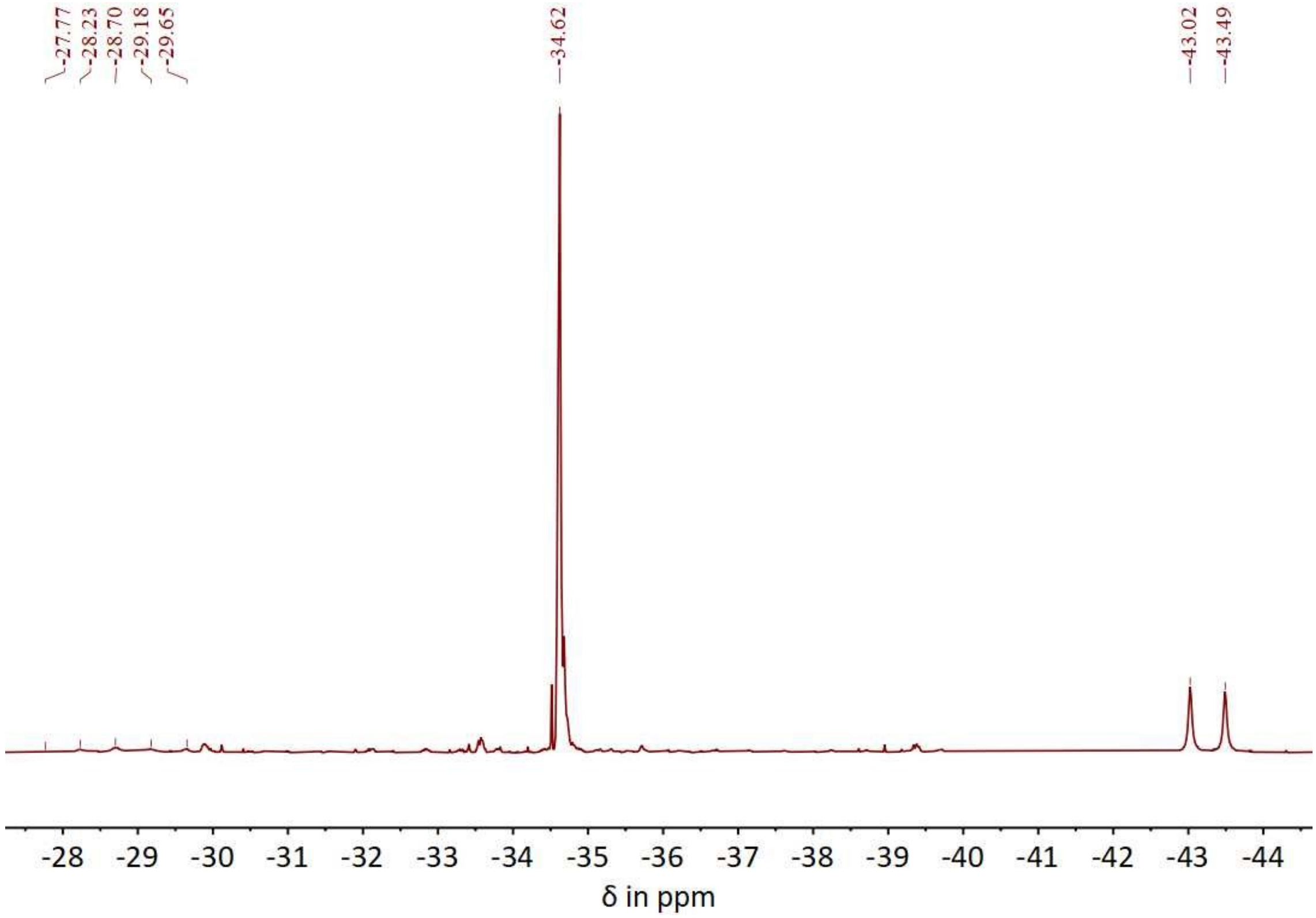

Figure S1 ^{19}F NMR spectrum (377 MHz, 22 °C, MeCN-d$_3$) of a mixture of $[Pt(MeCN)_4][Pt(OTeF_5)_6]$ and $[NEt_4][OTeF_5]$.

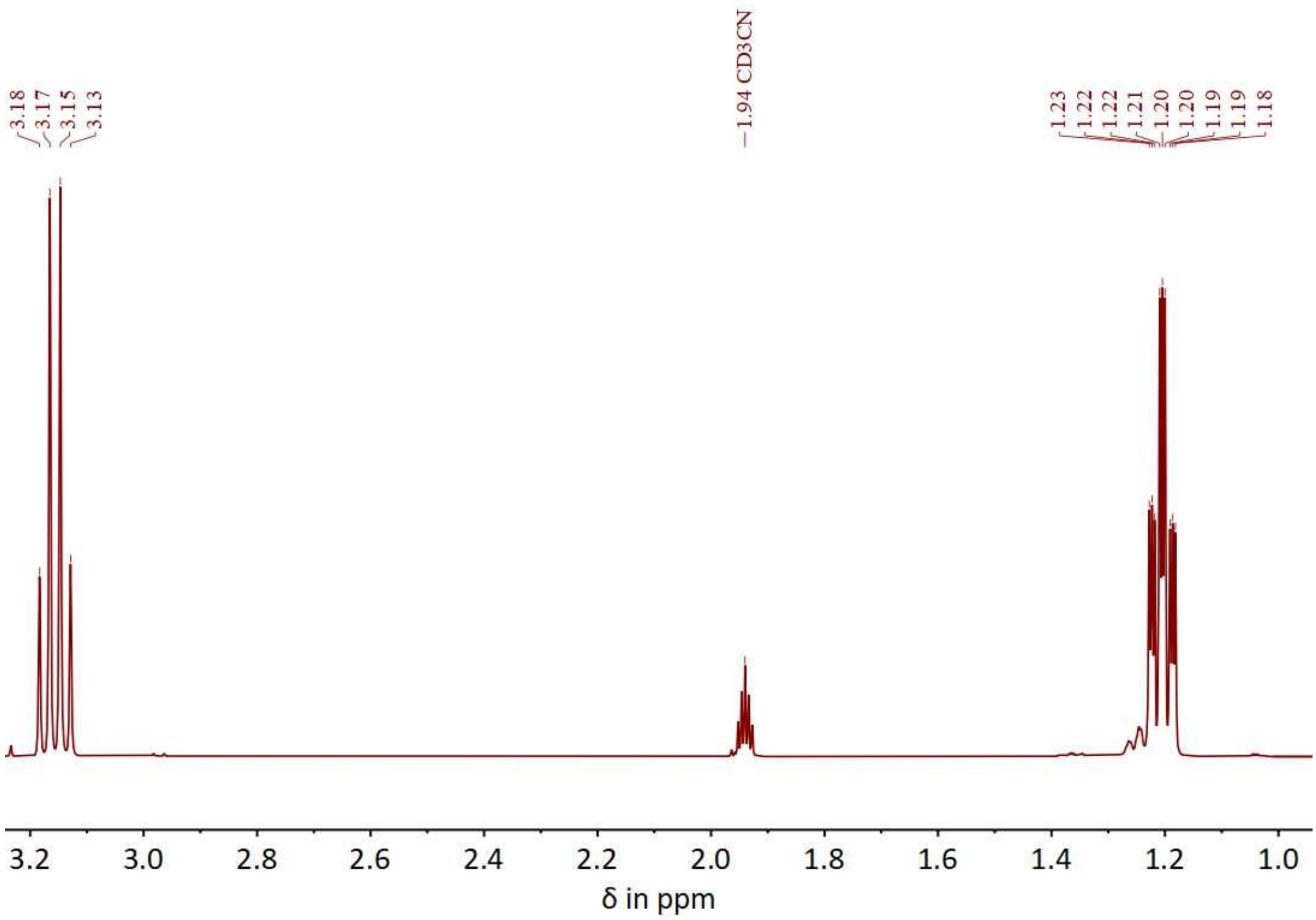

Figure S2 ^{1}H NMR spectrum (400 MHz, 22 °C, MeCN-d$_3$) of a mixture of $[Pt(MeCN)_4][Pt(OTeF_5)_6]$ and $[NEt_4][OTeF_5]$.

3 IR spectra

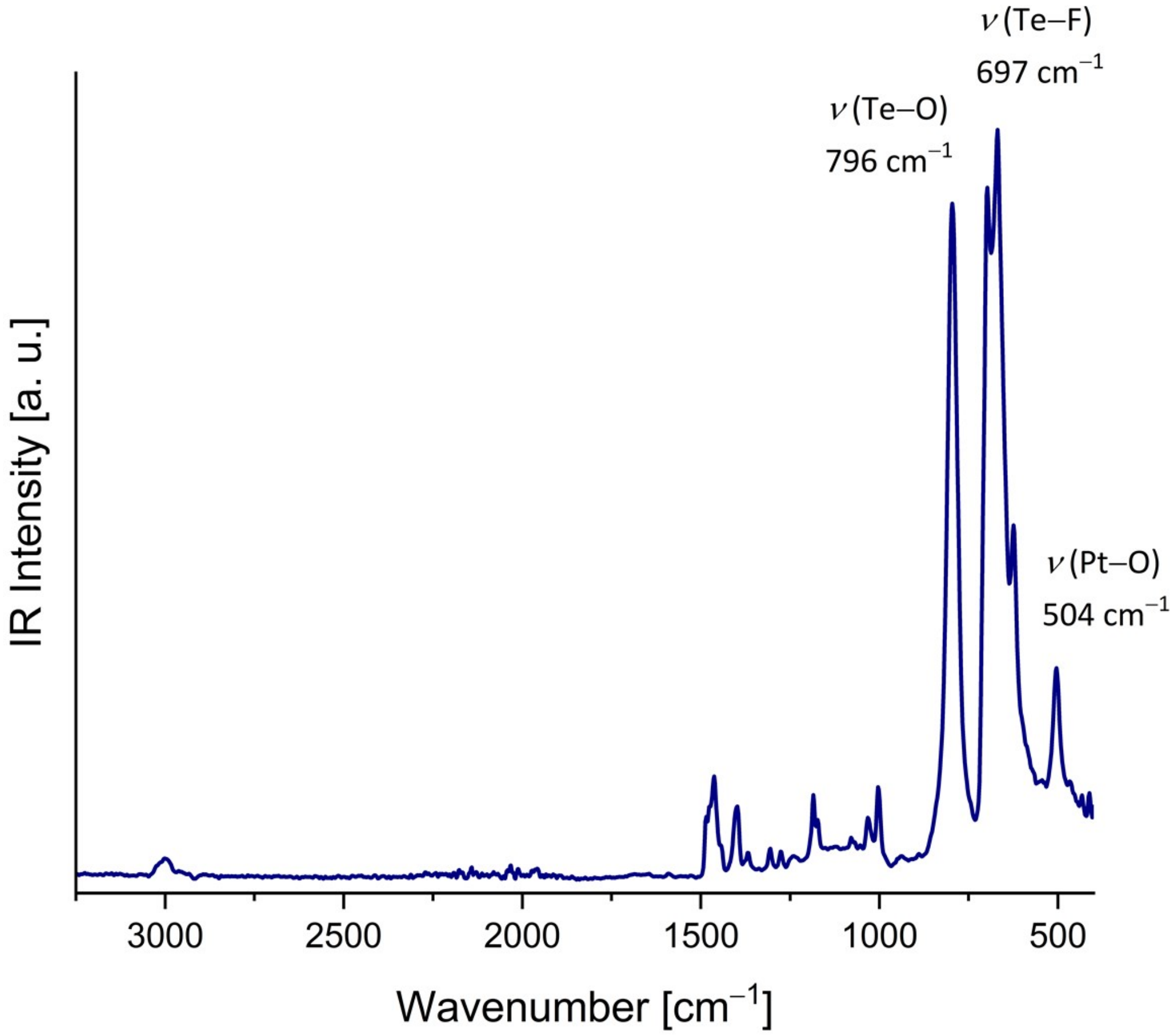

Figure S3 IR spectrum (ATR, 20 °C) of $[NEt_4]_2[Pt(OTeF_5)_6]$.

4 UV-VIS spectra

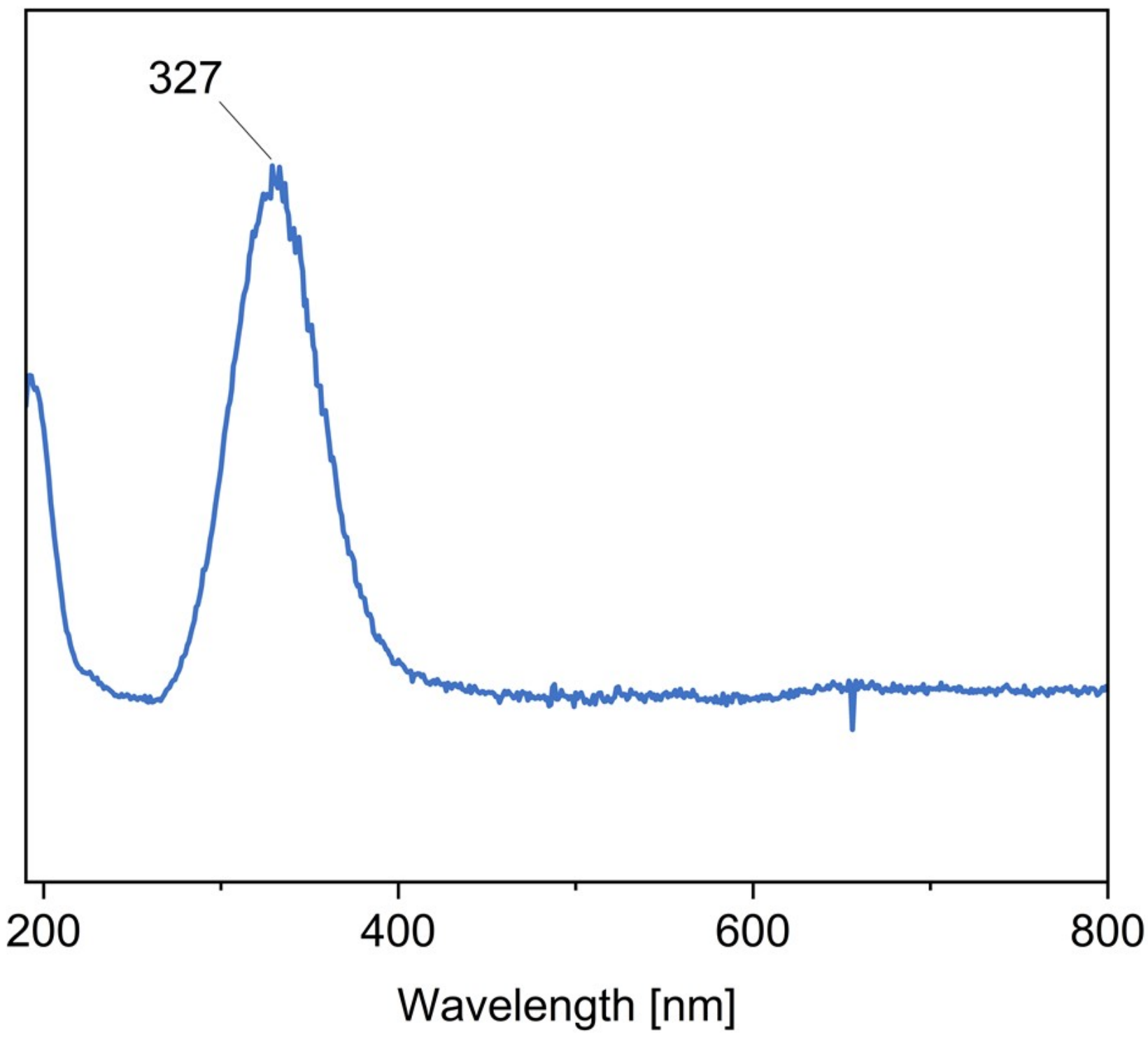

Figure S4 UV/Vis spectrum of the gas phase of the reaction between $[NEt_4]_2[PtCl_6]$ and $ClOTeF_5$, showing the formation of gaseous chlorine (327 nm).[15]

5 Thermogravimetric analysis (TGA)

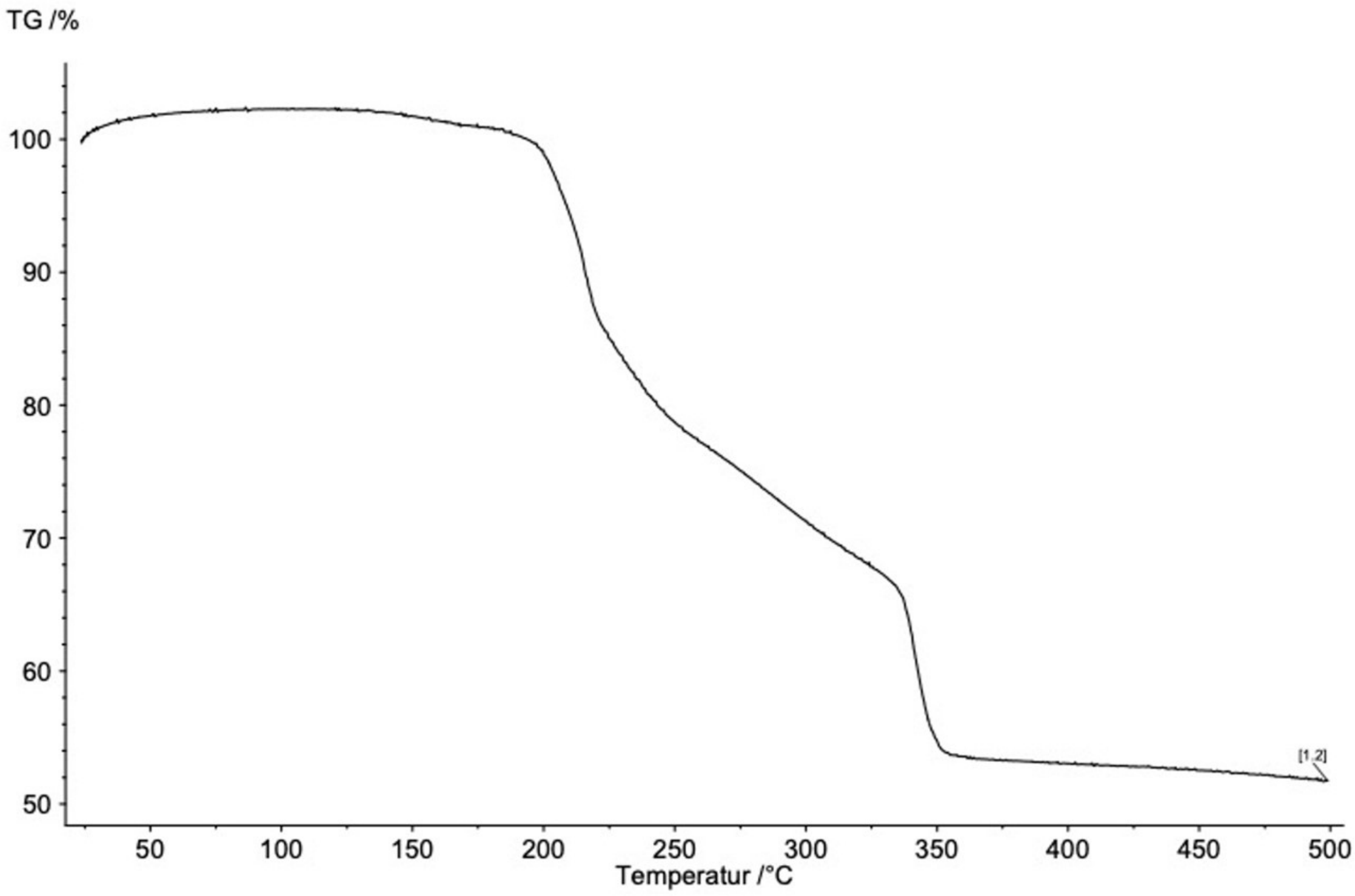

Figure S5 Thermogravimetric analysis of $[NEt_4]_2[Pt(OTeF_5)_6]$ recorded on heating at 5 K/min.

6 Crystal data

Table S1 Selected bond lengths of $[NEt_4]_2[Pt(OTeF_5)_6]$, where the asymmetric unit contains four crystallographically independent platinum centres, each of them coordinated by three pairs of symmetry-related teflate ligands.

Atom–Atom	Length [Å]	Atom–Atom	Length [Å]	Atom–Atom	Length [Å]
Pt1–O2	1.983(6)	Te2–F8	1.827(6)	Te7–F31	1.845(7)
Pt1–O3	1.997(7)	Te2–O2	1.838(6)	Te7–F32	1.826(6)
Pt1–O1	1.984(7)	Te4–F16	1.836(6)	Te7–F34	1.841(6)
Pt4–O11	2.000(7)	Te4–F20	1.844(6)	Te7–O7	1.831(7)
Pt4–O12	1.985(7)	Te4–F17	1.832(6)	Te11–F51	1.839(6)
Pt4–O10	1.980(7)	Te4–F18	1.833(6)	Te11–F53	1.849(6)
Pt2–O6	2.001(7)	Te4–F19	1.839(7)	Te11–F54	1.850(7)
Pt2–O4	1.995(7)	Te4–O4	1.823(7)	Te11–F55	1.830(7)
Pt2–O5	2.004(7)	Te6–F27	1.841(6)	Te11–O11	1.826(7)
Pt3–O8	1.996(7)	Te6–F28	1.846(7)	Te11–F52	1.820(7)
Pt3–O9	1.975(7)	Te6–F29	1.835(6)	Te12–F57	1.833(7)
Pt3–O7	1.983(7)	Te6–F30	1.816(6)	Te12–F60	1.834(7)
Te3–F13	1.847(6)	Te6–F26	1.840(6)	Te12–F56	1.836(7)
Te3–F12	1.840(6)	Te6–O6	1.826(7)	Te12–F58	1.845(7)
Te3–F15	1.837(6)	Te5–F21	1.839(6)	Te12–F59	1.819(7)
Te3–F11	1.831(6)	Te5–F23	1.833(6)	Te12–O12	1.825(7)
Te3–F14	1.819(6)	Te5–F22	1.843(7)	Te9–F44	1.843(6)
Te3–O3	1.832(7)	Te5–F24	1.825(7)	Te9–F41	1.837(7)
Te1–F5	1.845(6)	Te5–F25	1.837(6)	Te9–F43	1.825(7)
Te1–F4	1.835(6)	Te5–O5	1.822(7)	Te9–F45	1.832(7)
Te1–F1	1.843(6)	Te10–F46	1.852(7)	Te9–O9	1.836(7)
Te1–F2	1.840(6)	Te10–F47	1.835(7)	Te9–F42	1.818(8)
Te1–F3	1.827(6)	Te10–F49	1.834(7)	Te8–F39	1.840(7)
Te1–O1	1.829(7)	Te10–F48	1.838(7)	Te8–F38	1.850(7)
Te2–F9	1.838(6)	Te10–F50	1.822(7)	Te8–F37	1.839(6)
Te2–F10	1.838(6)	Te10–O10	1.827(7)	Te8–F36	1.845(7)
Te2–F6	1.844(6)	Te7–F35	1.832(6)	Te8–O8	1.832(7)
Te2–F7	1.829(6)	Te7–F33	1.839(7)	Te8–F40	1.817(7)

Table S2 Selected bond lengths of $[Pt(MeCN)_4][Pt(OTeF_5)_6]$.

Atom–Atom	Length [Å]	Atom–Atom	Length [Å]	Atom–Atom	Length [Å]
Pt2–O1	1.988(6)	Te2–F6	1.854(5)	C1–C2	1.456(12)
Pt2–O2	1.985(5)	Te2–F8	1.827(5)	N3–C5	1.140(15)
Pt2–O3	1.986(5)	Te2–O2	1.829(5)	C2–H2A	0.9600
Pt1–N2	1.953(7)	Te2–F9	1.831(5)	C2–H2B	0.9600
Pt1–N1	1.957(7)	Te1–O1	1.825(6)	C2–H2C	0.9600
Te3–F11	1.844(5)	Te1–F5	1.842(5)	C4–H4A	0.9600
Te3–F14	1.832(5)	Te1–F4	1.827(5)	C4–H4B	0.9600
Te3–F13	1.840(5)	Te1–F2	1.835(6)	C4–H4C	0.9600
Te3–F12	1.830(5)	Te1–F3	1.836(5)	C5–C6	1.456(17)

Te3–F15	1.831(5)	Te1–F1	1.836(5)	C6–H6A	0.9600
Te3–O3	1.837(5)	N2–C3	1.116(11)	C6–H6B	0.9600
Te2–F10	1.834(5)	N1–C1	1.121(11)	C6–H6C	0.9600
Te2–F7	1.830(5)	C3–C4	1.463(13)		

Table S3 Crystal data and structure refinement for compounds $[NEt_4]_2[Pt(OTeF_5)_6]$ and $[Pt(MeCN)_4][Pt(OTeF_5)_6]$.

CCDC number	2547134	2547135
Empirical formula	$C_{16}H_{40}F_{30}N_2O_6PtTe_6$	$C_{12}H_{18}F_{30}N_6O_6Pt_2Te_6$
Formula weight	1887.19	2068.10
Temperature [K]	100.00	100.00
Crystal system	triclinic	triclinic
Space group (number)	$P\bar{1}$	$P\bar{1}$
a [Å]	8.9262(5)	9.9368(7)
b [Å]	22.7657(12)	11.3267(8)
c [Å]	24.3043(13)	11.8309(8)
α [°]	62.164(2)	117.820(2)
β [°]	86.803(2)	96.192(3)
γ [°]	87.804(2)	107.787(2)
Volume [Å^3]	4360.2(4)	1070.65(13)
Z	4	1
ρ_{calc} [gcm^{-3}]	2.875	3.208
μ [mm^{-1}]	7.316	10.701
F(000)	3432	918
Crystal size [mm^3]	0.054×0.125×0.241	0.478×0.596×0.754
Crystal colour	clear yellow	clear yellow
Crystal shape	plate	block
Radiation	MoK_α (λ=0.71073 Å)	MoK_α (λ=0.71073 Å)
2θ range [°]	4.57 to 50.70 (0.83 Å)	4.08 to 50.73 (0.83 Å)
Index ranges	−10 ≤ h ≤ 10<br>−23 ≤ k ≤ 27<br>0 ≤ l ≤ 29	−11 ≤ h ≤ 11<br>−13 ≤ k ≤ 13<br>−14 ≤ l ≤ 14
Reflections collected	16564	48003
Independent reflections	16564<br>R_{int} = 0.0594<br>R_{sigma} = 0.0248	3925<br>R_{int} = 0.0649<br>R_{sigma} = 0.0254
Completeness to θ = 25.242°	99.1 %	100.0 %
Data / Restraints / Parameters	16564 / 1 / 1082	3925 / 0 / 287
Absorption correction T_{min}/T_{max} (method)	0.4726 / 0.7454<br>(multi-scan)	0.0053 / 0.0390<br>(multi-scan)
Goodness-of-fit on F^2	1.129	1.202
Final *R* indexes [I≥2σ(I)]	R_1 = 0.0321<br>wR_2 = 0.0725	R_1 = 0.0363<br>wR_2 = 0.0942
Final *R* indexes [all data]	R_1 = 0.0425<br>wR_2 = 0.0809	R_1 = 0.0369<br>wR_2 = 0.0946
Largest peak/hole [eÅ^{-3}]	1.44/−1.41	2.47/−2.17

7 Quantum-chemical Analyses

$$[NEt_4]_2[PtCl_x(OTeF_5)_{6-x}] + ClOTeF_5 \xrightarrow{\Delta_r G} [NEt_4]_2[PtCl_{x-1}(OTeF_5)_{7-x}] + Cl_2$$

Scheme S1 Reaction scheme of the stepwise introduction of the teflate ligand to the platinum centre.

Table S4 Gibbs free energies of the stepwise introduction of the teflate ligand to the platinum centre based of quantum chemical calculation on the B3LYP/def2-TZVPPD niveau.

x	Product	$\Delta_r G$ (kJ/mol), B3LYP/def2-TZVPPD	$\Delta_r H$ (kJ/mol), B3LYP/def2-TZVPPD
6	$[PtCl_5(OTeF_5)]^{2-}$	–135.58	–147.61
5	*cis*-$[PtCl_4(OTeF_5)_2]^{2-}$	–121.45	–134.11
4	*fac*-$[PtCl_3(OTeF_5)_3]^{2-}$	–104.79	–121.76
3	*cis*-$[PtCl_2(OTeF_5)_4]^{2-}$	–89.79	–103.33
2	$[PtCl(OTeF_5)_5]^{2-}$	–80.85	–101.04
1	$[Pt(OTeF_5)_6]^{2-}$	–80.20	–101.11

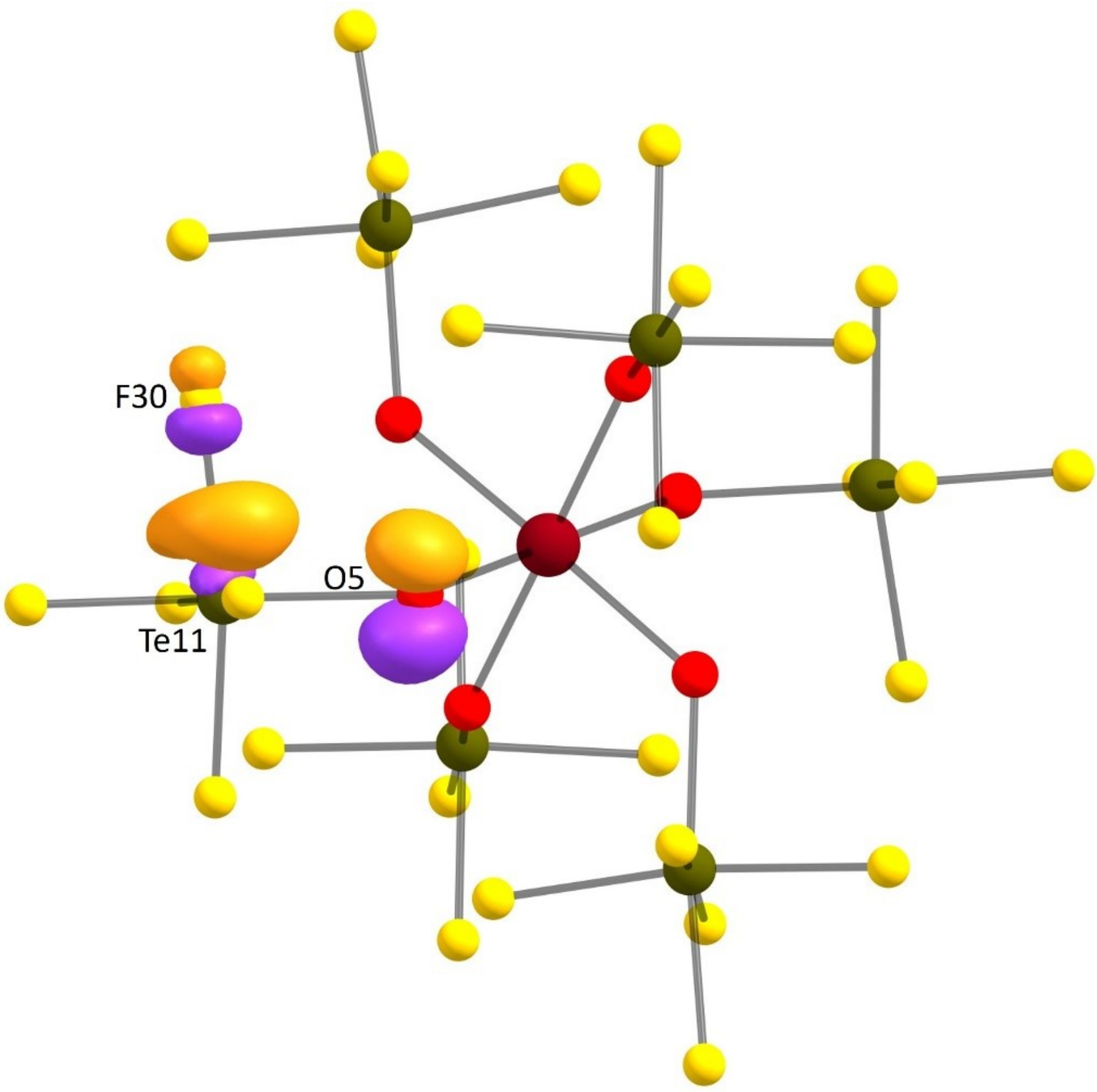

Figure S6 Negative hyperconjugation from NBO 106 (p-type lone pair at O5) into NBO 260 (σ*(Te11–F30) antibonding orbital), resulting in a stabilization energy of 37.9 kJ/mol.

8 EDA-NOCV Analysis

Table S5 Calculated EDA-NOCV energies in kcal/mol for $[Ti(OTeF_5)_6]^{2-}$ and $[Pt(OTeF_5)_6]^{2-}$ (B3LYP/def2-TZVPPD). %E_{orb} represents the percentage of the part of E_{orb} out of E_{orb}+E_{elstat}

	E_{int}	E_{orb}	E_{elstat}	E_{pauli}	E_{steric}	E_{disp}	%E_{orb}
$[Ti(OTeF_5)_6]^{2-}$	−103	−68	−52	134	−29	−87	57%
$[Pt(OTeF_5)_6]^{2-}$	−122	−109	−62	196	−49	−98	64%

$[Pt(OTeF_5)_6]^{2-}$ $[Ti(OTeF_5)_6]^{2-}$

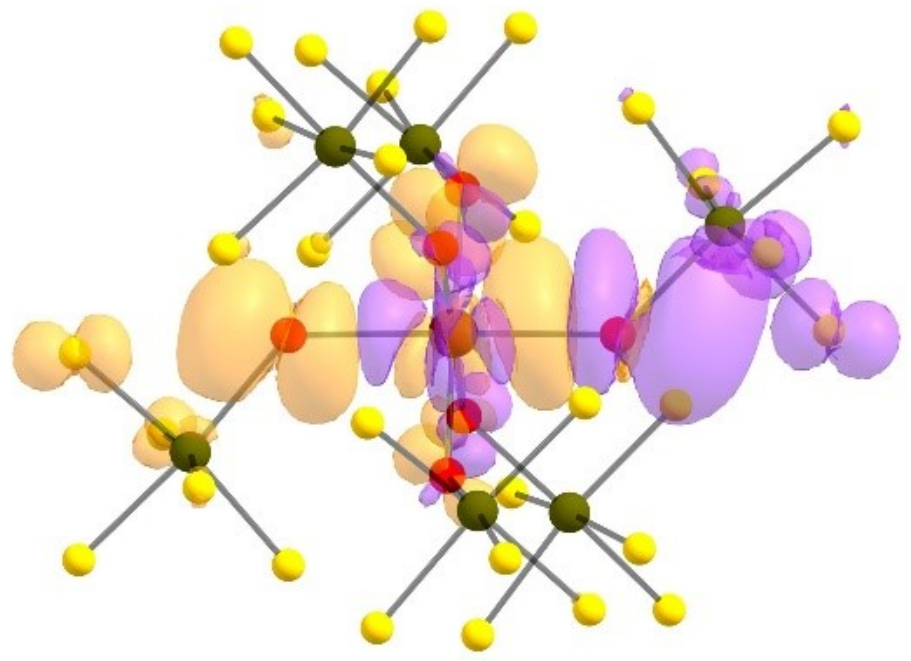

$\Delta\rho_{(1)}$: $E_{orb(1)}$ = −79 kcal·mol^{-1}; $|v_1|$ = 0.79

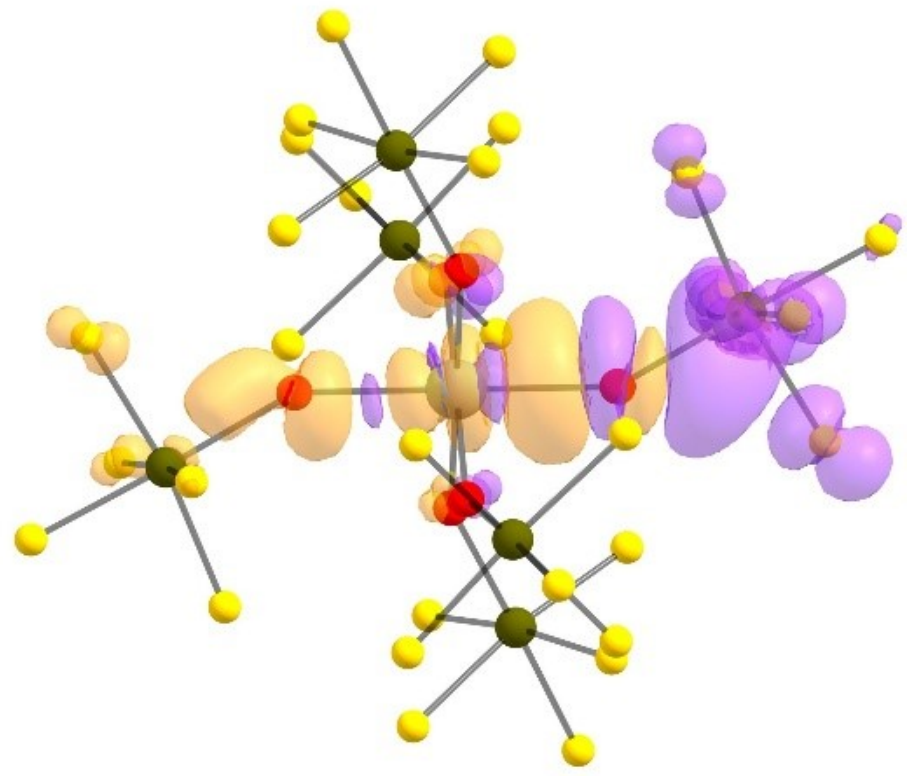

$\Delta\rho_{(1)}$: $E_{orb(1)}$ = −32 kcal·mol^{-1}; $|v_1|$ = 0.51

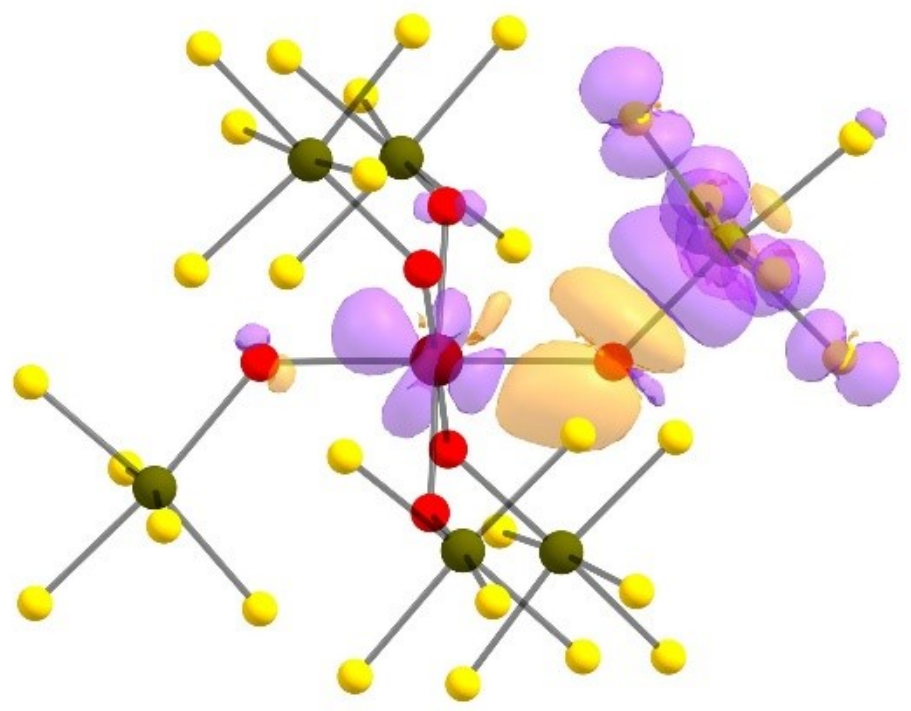

$\Delta\rho_{(2)}$: $E_{orb(2)}$ = −9 kcal·mol^{-1}; $|v_2|$ = 0.26

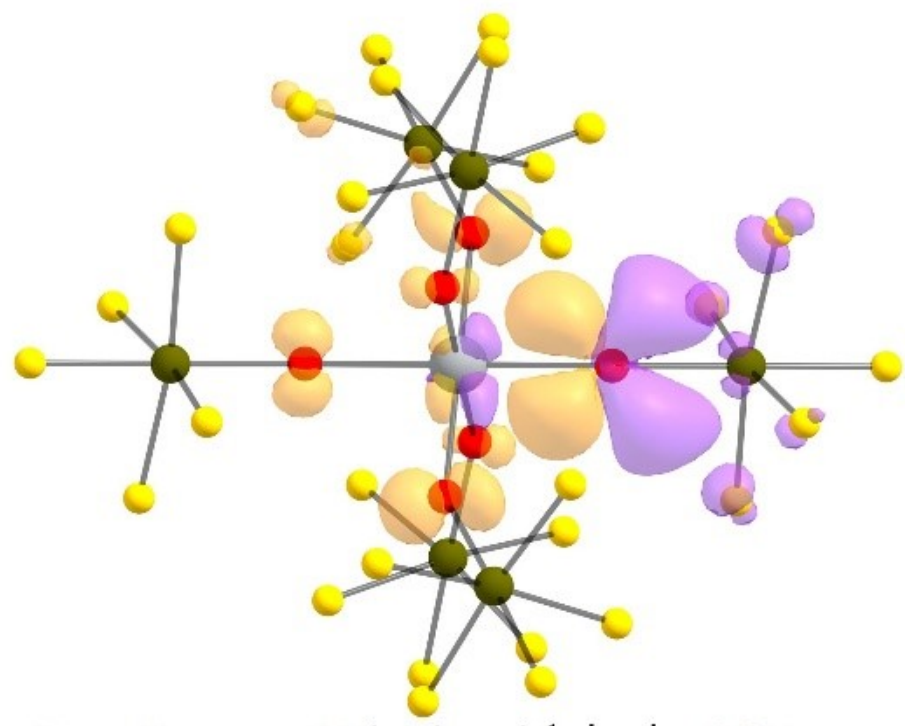

$\Delta\rho_{(2)}$: $E_{orb(2)}$ = −11 kcal·mol^{-1}; $|v_2|$ = 0.37

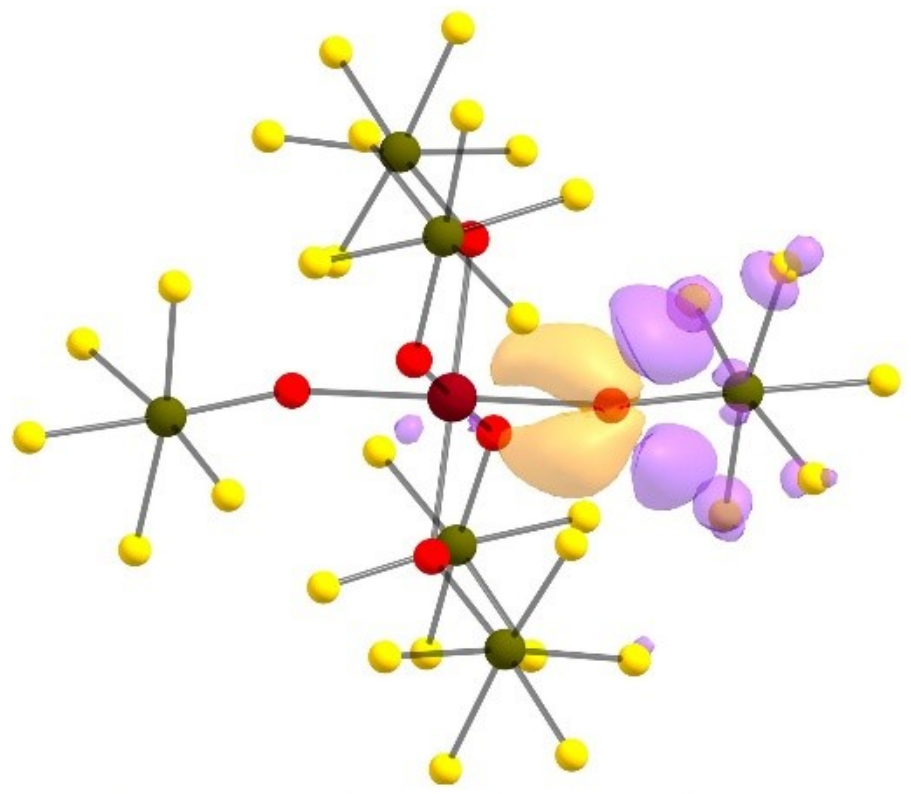

$\Delta\rho_{(3)}$: $E_{orb(3)}$ = −5 kcal·mol^{-1}; $|v_3|$ = 0.15

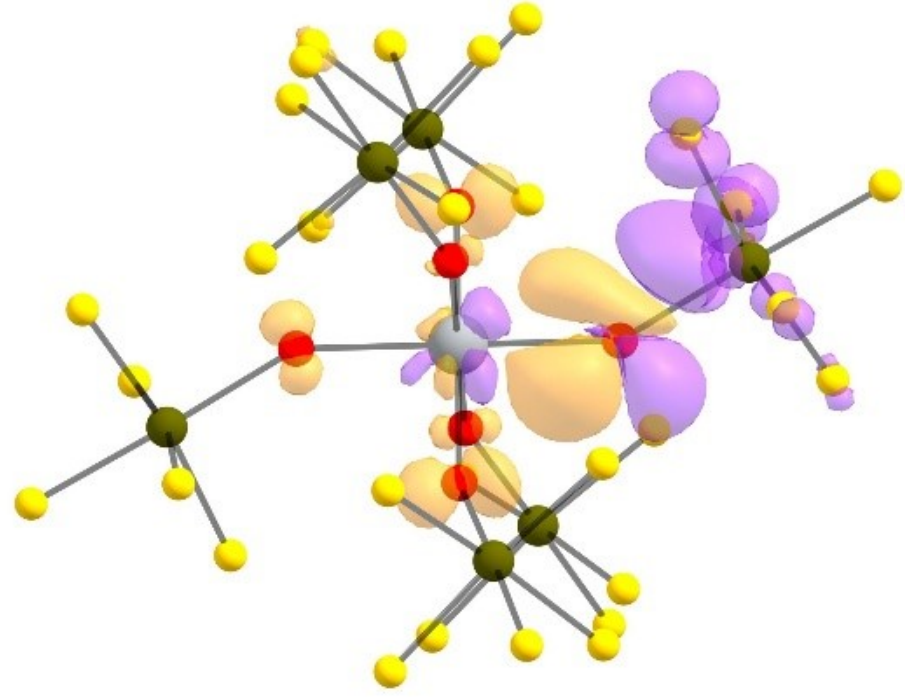

$\Delta\rho_{(3)}$: $E_{orb(3)}$ = −11 kcal·mol^{-1}; $|v_3|$ = 0.34

Figure S7 Deformation density plots for $[Pt(OTeF_5)_6]^{2-}$ (left) and $[Ti(OTeF_5)_6]^{2-}$ (right) of NOCVs (natural orbitals for chemical valence) 1-3 (B3LYP-B3BJ/TZVPPD). The isosurface value for the plots is 0.001 au. Charge flow direction in the deformation densities is from orange to purple.

9 OSLO Analysis

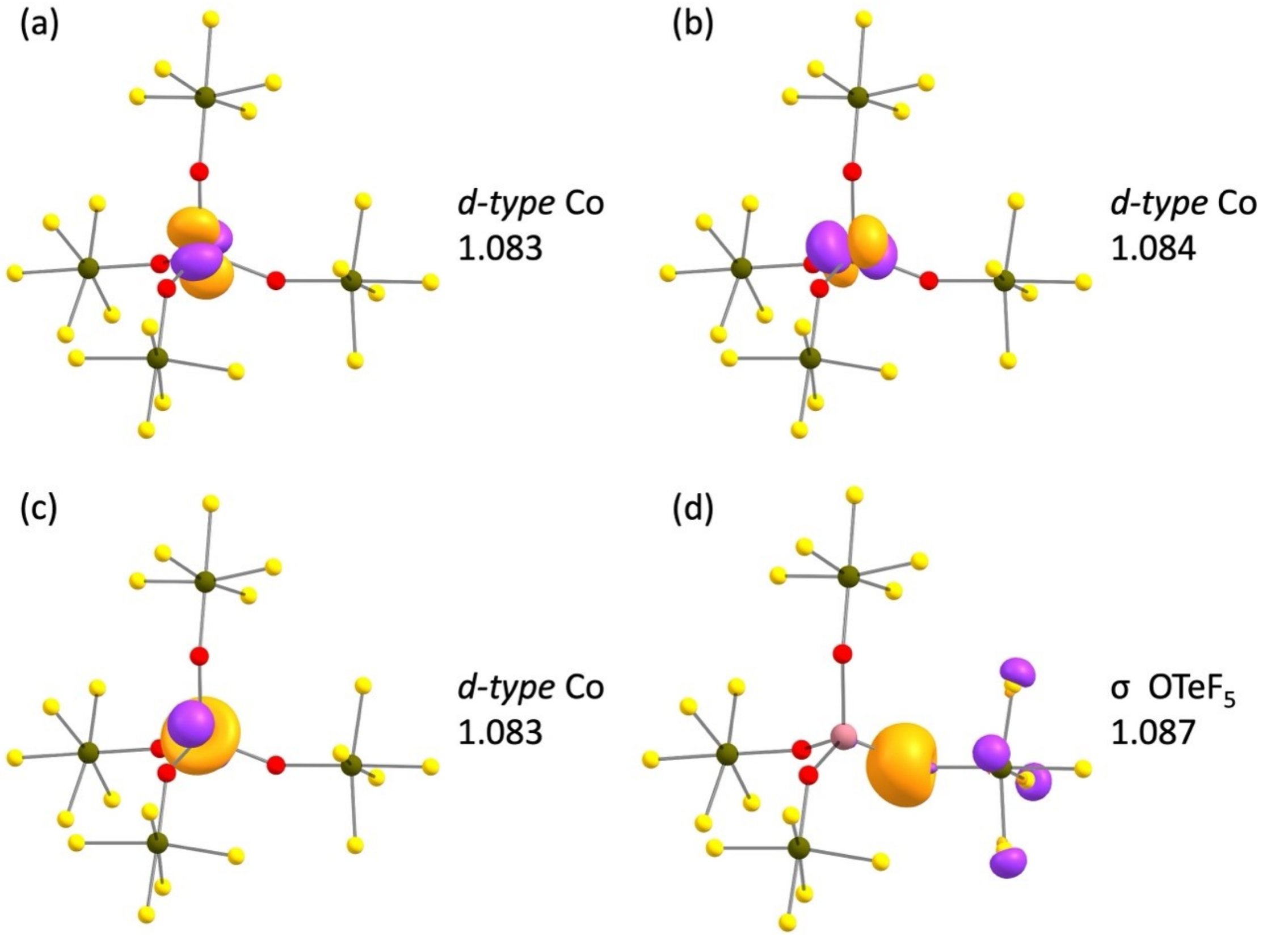

Figure S8 Selected OSLOs of $[Co(OTeF_5)_4]^{2-}$ with TFVC FOLI values for three d-type orbitals at Co (a)-(c) and the $OTeF_5$ ligand (d). The polar character of the σ Co–$OTeF_5$ interaction is clearly evident. The FOLI values, as well as the visual inspection lead to a clear assignment of the respective orbital to either the Co centre or the ligand. The isosurface value for the plots is 0.075 au.

Table S6 Summary of the OSLO analysis together with the corresponding experimental mean bond lengths for each compound, as determined by X-ray diffraction studies. The molecular structures were optimized at the B3LYP/def2-TZVPPD level, and the OSLO results were obtained using TFVC population analysis.

Compound	CCDC	M–O Mean [Å]	O–Te Mean [Å]	Te–F Mean [Å]	M–O–Te Mean [°]	FOLI of last OSLO	ΔFOLI of last OSLO	FOLI of σ bond Pt-O
$[Mn(OTeF_5)_4]^{2-}$	2287496	2.020	1.764	1.838	134.0	1.103	3.177	1.083
$Ag_2(OTeF_5)_2$·2DCE	1181486	2.337	1.781	1.845	129.3	1.092	4.883	1.092
$[Co(OTeF_5)_4]^{2-}$	2251373	1.948	1.788	1.848	133.6	1.118	6.909	1.083
$Al(OTeF_5)_4^-$	1971173	1.750	1.790	1.828	139.9	1.095	6.413	1.095
$[Ni(OTeF_5)_4]^{2-}$	2171818	1.946	1.796	1.842	130.0	1.118	3.040	1.118
$[Hg(OTeF_5)_4]^{2-}$	1055055	2.217	1.799	1.844	121.4	1.136	2.809	1.136
$[Ti(OTeF_5)_6]^{2-}$	1202467	1.936	1.816	1.840	143.4	1.149	4.912	1.149
$Nb(OTeF_5)_6^-$	1308724	1.930	1.820	1.810	154.7	1.182	3.108	1.182
$[Pd(OTeF_5)_4]^{2-}$	1194845	2.002	1.826	1.838	124.3	1.183	3.982	1.183
$[Pt(OTeF_5)_6]^{2-}$	2547134	1.990	1.829	1.838	131.7	1.348	1.623	1.348
$B(OTeF_5)_4^-$	654408	1.471	1.831	1.830	130.0	1.139	6.376	1.139
$[Au(OTeF_5)_4]^{2-}$	2156160	1.973	1.840	1.829	120.9	1.358	2.106	1.358
$Sb(OTeF_5)_6^-$	2453010	1.953	1.843	1.827	139.0	1.250	3.331	1.250

$Bi(OTeF_5)_6^-$	1292863	2.057	1.851	1.820	136.0	1.327	1.738	1.327
$Te(OTeF_5)_6$	1592104	1.904	1.889	1.817	139.1	1.345	2.272	1.345
$As(OTeF_5)_6^-$	1292864	1.780	1.890	1.800	140.5	1.274	3.391	1.274
$C(OTeF_5)_4$	654407	1.370	1.894	1.820	131.0	1.339	3.926	1.339

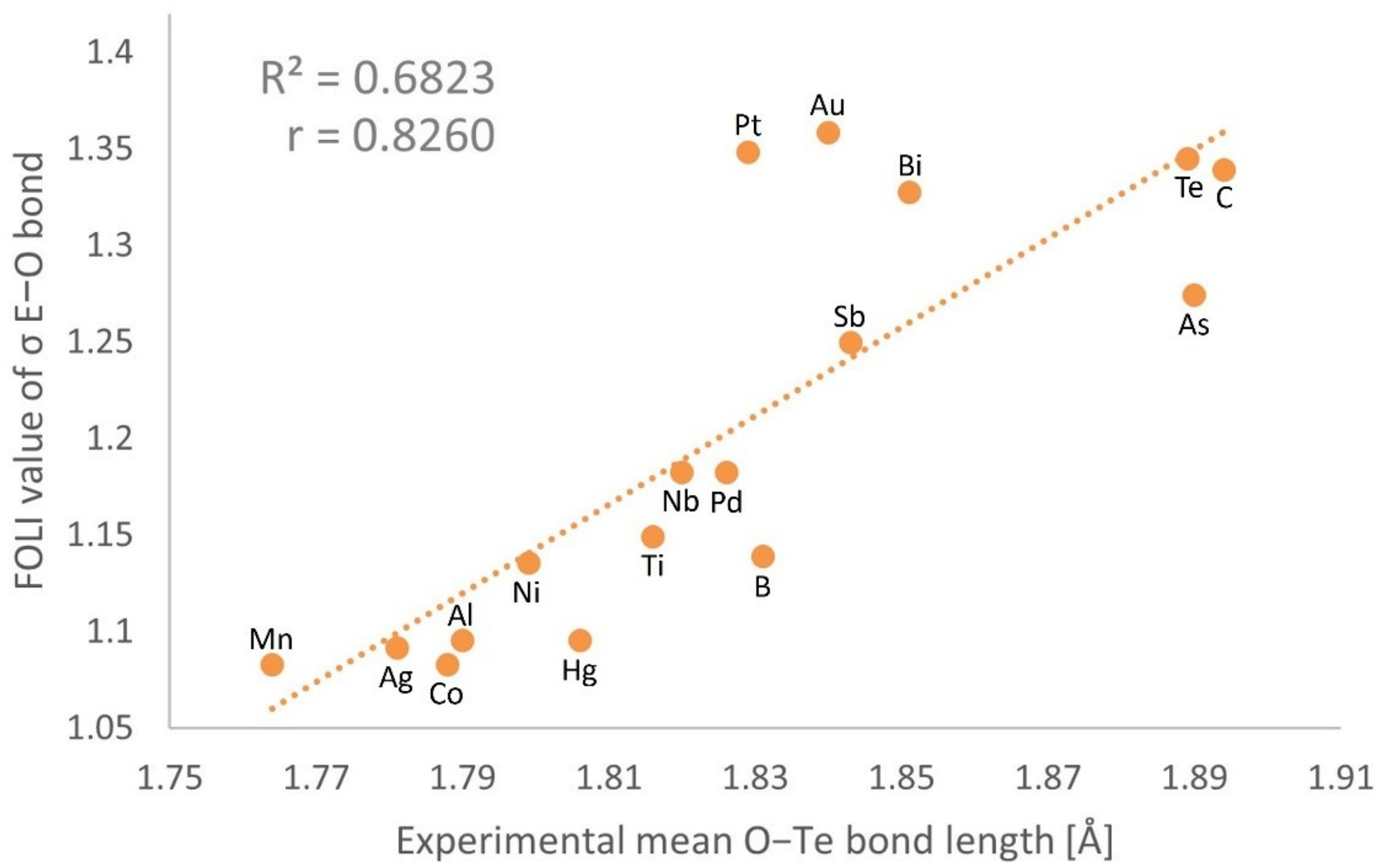

Figure S9 Correlation of the experimental mean bond lengths of the O–Te bond with the FOLI value of the σ E–O bond.

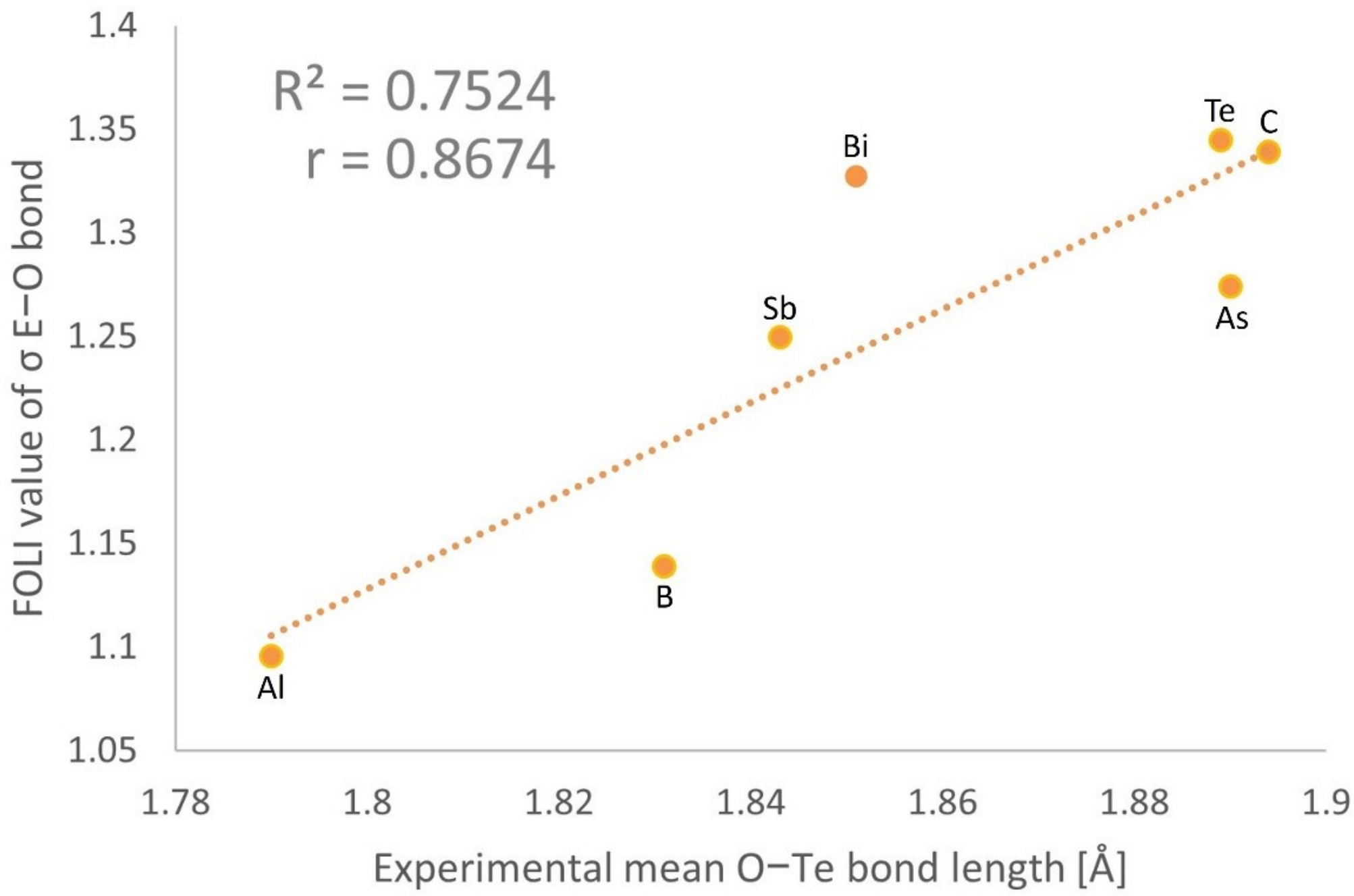

Figure S10 Correlation of the experimental mean bond lengths of the O–Te bond with the FOLI value of the σ E–O bond.

Examplary OSLO Input File for $[Pt(OTeF_5)_6]^{2-}$:

```
# METHOD
TFVC
OSLO
MOKIT
DOFRAGS
#
# OSLO
TFVC
#
# FRAGMENTS
7
1
1
7
2 8 14 15 16 17 18
7
3 9 19 20 21 22 23
7
4 10 24 25 26 27 28
7
5 11 29 30 31 32 33
7
6 12 34 35 36 37 38
7
7 13 39 40 41 42 43
```

10 NPA Charges and QTAIM

Table S7 QTAIM parameters at the Pt/Ti–O and O–Te bond critical points of $[M(OTeF_5)_6]^{2-}$ (M = Pt, Ti). Listed are the mean values of electron density ρ(r), Laplacian of the electron density $\nabla^2\rho(r)$, total energy density H(r), kinetic energy density G(r), potential energy density V(r), electron localization function (ELF), the |V|/G ratio, and the bond ellipticity ε. All QTAIM quantities are given in atomic units.

Compound	ρ(r)	∇²ρ(r)	H(r)	G(r)	V(r)	ELF	|V|/G	ε
$[Pt(OTeF_5)_6]^{2-}$ Pt−O	0.097	0.454	−0.013	0.127	−0.140	0.179	1.104	0.034
$[Pt(OTeF_5)_6]^{2-}$ O−Te	0.178	0.536	−0.098	0.232	−0.329	0.328	1.421	0.006
$[Ti(OTeF_5)_6]^{2-}$ Ti−O	0.123	0.456	−0.042	0.153	−0.195	0.244	1.276	0.021
$[Ti(OTeF_5)_6]^{2-}$ O−Te	0.171	0.457	−0.093	0.207	−0.300	0.348	1.449	0.064

Table S8 Comparison of NPA and QTAIM charges in $[M(OTeF_5)_6]^{2-}$ (M = Pt, Ti). Mean atomic charges are reported for M, O, Te, and F, together with the mean summed charge of $-OTeF_5$ units.

Compound	Type	M	O	Te	F	$OTeF_5$
$[Pt(OTeF_5)_6]^{2-}$	NPA	1.79	−1.02	3.39	−0.60	−0.63
	QTAIM	1.81	−1.00	3.65	−0.66	−0.64
$[Ti(OTeF_5)_6]^{2-}$	NPA	1.62	−1.01	3.41	−0.60	−0.60

	QTAIM	2.48	−1.14	3.68	−0.66	−0.75

11 References

1 S. Fundel, Dissertation, Freie Universität Berlin, 2003.
2 M. Jankowska-Wajda, O. Bartlewicz, A. Walczak, A. R. Stefankiewicz and H. Maciejewski, *J. Catal.*, 2019, **374**, 266.
3 R. K. Harris, E. D. Becker, S. M. Cabral de Menezes, P. Granger, R. E. Hoffman and K. W. Zilm, *Magnetic resonance in chemistry*, 2008, **46**, 582.
4 Adept Scientific, gNMR V 5.0, 2005.
5 G. M. Sheldrick, *Acta Cryst. A*, 2015, **71**, 3.
6 G. M. Sheldrick, *Acta Cryst. C*, 2015, **71**, 3.
7 O. V. Dolomanov, L. J. Bourhis, R. J. Gildea, J. A. K. Howard and H. Puschmann, *Journal of Applied Crystallography*, 2009, **42**, 339.
8 F. Neese, *WIREs Comput. Mol. Sci.*, 2012, **2**, 73.
9 A. D. Becke, *Phys. Rev. A*, 1988, **38**, 3098.
10 F. Weigend and R. Ahlrichs, *Phys. Chem. Chem. Phys.*, 2005, **7**, 3297.
11 M. Gimferrer, A. Aldossary, P. Salvador and M. Head-Gordon, *J. Chem. Theory Comput.*, 2022, **18**, 309.
12 P. Salvador, E. Ramos-Cordoba, M. Montilla, L. Pujal and M. Gimferrer, *J. Chem. Phys.*, 2024, **160**, 175502.
13 P. Salvador and E. Ramos-Cordoba, *J. Chem. Phys.*, 2013, **139**, 71103.
14 a) T. Lu, F. Chen, *J. Comput. Chem*., 2012, **33**, 580; b) T. Lu, *J. Chem. Phys.*, 2024, **161**, 082503.
15 D. Maric, J. P. Burrows, R. Meller and G. K. Moortgat, *J. Photochem. Photobiol., A*, 1993, **70**, 205.
